# MZ1 co-operates with trastuzumab in HER2 positive breast cancer

**DOI:** 10.1186/s13046-021-01907-9

**Published:** 2021-03-19

**Authors:** María del Mar Noblejas-López, Cristina Nieto-Jiménez, Eva M. Galán-Moya, David Tebar-García, Juan Carlos Montero, Atanasio Pandiella, Miguel Burgos, Alberto Ocaña

**Affiliations:** 1 Translational Research Unit, Translational Oncology Laboratory, Albacete University Hospital, C/Francisco Javier de Moya esquina C/Laurel, Albacete, Spain; 2 Centro Regional de Investigaciones Biomédicas, Castilla-La Mancha University (CRIB-UCLM), Albacete, Spain; 3 Instituto de Biología Molecular y Celular del Cáncer (IBMCC-CIC), Salamanca, Spain; 4 Faculty of Nursing, Castilla-La Mancha University (UCLM), Albacete, Spain; 5 Instituto de Investigación Biomédica de Salamanca (IBSAL), Salamanca, Spain; 6 CIBERONC, Salamanca, Spain; 7 Consejo Superior de Investigaciones Científicas (CSIC), Salamanca, Spain; 8 Experimental Therapeutics Unit, Medical Oncology Department, Hospital Clínico San Carlos (HCSC), Instituto de Investigación Sanitaria (IdISSC) and CIBERONC, Calle Del Prof Martín Lagos, s/n, 28040 Madrid, Spain

**Keywords:** HER2+ breast cancer, ERBB2, Trastuzumab, PROTACs, MZ1

## Abstract

**Background:**

Although the anti-HER2 antibody trastuzumab augments patient survival in HER2+ breast cancer, a relevant number of patients progress to this treatment. In this context, novel drug combinations are needed to increase its antitumor activity. In this work, we have evaluated the efficacy of proteolysis targeting chimera (PROTAC) compounds based on BET inhibitors (BETi) to augment the activity of trastuzumab in HER2+ breast cancer models.

**Methods:**

BT474 and SKBR3 HER2+ breast cancer cell lines were used. The effects of trastuzumab and the BET-PROTAC MZ1 either alone or in combination, were evaluated using MTT proliferation assays, three-dimensional invasion and adhesion cultures, flow cytometry, qPCR and Western blot. In vivo studies were carried out in a xenografted model in mice. Finally, a Clariom_S_Human transcriptomic array was applied to identify deregulated genes after treatments.

**Results:**

MZ1 induced a higher antiproliferative effect compared to the BETi JQ1. The combination of MZ1 and -trastuzumab significantly decreased cell proliferation, the formation of three-dimensional structures and cellular invasion compared to either of the drugs alone. Evaluation of apoptosis resulted in an increase of cell death following treatment with the combination, and biochemical studies displayed modifications of apoptosis and DNA damage components. In vivo administration of agents alone or combined, to tumors orthotopically xenografted in mice, resulted in a decrease of the tumor volume only after MZ1-Trastuzumab combination treatment. Results from a transcriptomic array indicated a series of newly described transcription factors including HOXB7, MEIS2, TCERG1, and DNAJC2, that were associated to poor outcome in HER2+ breast cancer subtype and downregulated by the MZ1-trastuzumab combination.

**Conclusions:**

We describe an active novel combination that includes the BET-PROTAC MZ1 and trastuzumab, in HER2+ tumors. Further studies should be performed to confirm these findings and pave the way for their future clinical development.

**Supplementary Information:**

The online version contains supplementary material available at 10.1186/s13046-021-01907-9.

## Background

The HER2+ breast cancer subtype is a leading cause of female mortality in developed countries, accounting for around 20% of all diagnosed breast tumors [1]. The overexpression of the transmembrane tyrosine kinase HER2 in this subtype promotes an oncogenic phenotype that leads to the activation of downstream signaling pathways inducing proliferation and pro-survival signals [2]. In this line, the development of anti-HER2-targeted therapies, such as the monoclonal antibody trastuzumab, has demonstrated antitumoral activity improving clinical outcome in patients with HER2+ tumors. Novel strategies using trastuzumab as a backbone, like the antibody-drug conjugate trastuzumab-emtansine (T-DM1), have increased patient survival reverting resistance to trastuzumab [3]. In this context, the identification of novel therapeutics inducing antitumor activity in HER2 + tumors is a main goal with relevant clinical implications.

The acetylated lysine readers bromo and extra terminal domains (BET) family of proteins have been involved in several oncogenic processes in many different tumor types [4]. BET inhibitors (BETi) reduce the expression of oncogenic transcription factors and have been described as potential drugs to treat breast cancer [5, 6]. Compounds blocking the activity of the BET family member BRD4 have gained attention due to their preclinical activity, and have now reached early stage clinical development [7]. In the last years, novel agents named PROTACs (PROteolysis TArgeting Chimeras) have been engineered to target specific proteins for ubiquitination and degradation through the proteasome machinery, demonstrating antitumoral activity [8, 9]. This effect leads to a complete degradation and therefore lack of protein activity. Among them, we find of particular interest those based on BETi structures, such us MZ1 [10], as they have shown to be highly efficient in different tumors including the triple negative breast cancer subtype [11, 12].

Identification of synergistic combinations that increase the antitumor activity is a key aim to optimize anti-HER2 cancer therapeutics. In this context, different molecular vulnerabilities where a therapeutic intervention could potentially augment the antitumor effect of trastuzumab have been identified. In line with this, some of those therapies have already shown efficacy in patients, while others are still in preclinical development [13–15]. Trastuzumab has shown a synergistic effect with different therapeutics, for instance in combination with pertuzumab, an anti-HER2 antibody designed against a different epitope located in the HER2 dimerization arm [16]; small tyrosine kinase inhibitors like lapatinib [17]; or approved chemotherapies like taxanes [18]. Alternative approaches are still in early clinical or preclinical evaluation as the inhibition of intracellular mediators, like the SRC kinase, or the combination with compounds that induce apoptotic mechanisms, among others [19, 20].

Previous data generated in our laboratory suggest that the expression of some bromodomain transcripts is associated with detrimental prognosis in patients with HER2+ tumors [21]. With this background in mind, in the present study we aimed to explore the potential synergistic action between trastuzumab and the BET-PROTAC inhibitor MZ1 in HER2 overexpressing breast cancer cells. We observed that the administration of MZ1 augmented the antiproliferative capacity of trastuzumab as a single agent, mainly by inducing DNA damage and apoptosis. This result was confirmed in several cell lines and in an in vivo model. Gene expression analysis identified the “regulation of transcription” as a key modulated function, recognizing transcription factors that were associated with detrimental prognosis. Altogether, we describe the augmented effect of the combination of trastuzumab and the BET-PROTAC MZ1, paving the way for their future evaluation in HER2+ breast cancer patients.

## Methods

### Cell lines culture and drugs

Breast cancer HER2+ cells BT474 and SKBR3 were cultured in DMEM (Sigma-Aldrich, Saint Louis, MO, USA) supplemented with inactivated fetal bovine serum (10%), antibiotics (100 U/mL penicillin and 100 U/mL streptomycin) (37 °C, 5% CO_2_). MCF10A cells were maintained in DMEM/F12 supplemented with 5% horse serum, 20 ng/mL EGF, 0.5 μg/mL hydrocortisone, 10 μg/mL insulin, 1% non-essential amino acids and antibiotics. BRD4-PROTACs MZ1, ARV-825 and Cis-MZ1 (inactive form of MZ1) were purchased from Tocris Bioscience BET inhibitors JQ1 and OTX-015 were purchased from Selleckchem (Houston, TX). Trastuzumab were purchased as Herceptin (ROCHE).

### Proliferation assay (MTT)

Anti-proliferative effect was evaluated through MTT (Sigma Aldrich) colorimetric assay. Cells were seeded on 48-wells plates and were treated 24 h later with indicated doses of each drug, alone or in combination. Cell medium was replaced at 72 h with MTT solution (red phenol-free DMEM with MTT 0.5 μg/μL) for 45 min at 37 °C. DMSO was then used to solubilize the samples. Absorbances at 555 nm values were recorded in a spectrophotometer multiwell plate reader. A reference wavelength of 690 nm was used.

To analyze if the effect of the compounds was additive or synergic, we combined different doses of MZ1 and trastuzumab and we used the Calcusyn 2.0 software (Biosoft). The mathematical expression used is the Chou-Talalay algorithm, which allows to obtain the combination index (CI) indicating synergistic effect (< 1), additive effect (=1) and antagonistic effect (> 1).

### Matrigel-3D invasion assay

Matrigel (Sigma-Aldrich) generates a tridimensional net that mimics the extracellular matrix. It allows evaluating the drug effect of invasiveness. Invading 3D structures were evaluated using an inverted microscope and sphere diameter was quantified using ImageJ software.

### Adhesion assay

BT474 treated for 24 h were recollected and seeded in fibronectin coated plates. Fibronectin (10 μg/mL, PBS, 1 h, 37 °C) was removed and plates were blocked (DMEM, 0,5% BSA, 45 min) and washed (DMEM 0,1% BSA). After cold shock, treated BT474 cells were reseeded. One hour later medium was removed, and adhered cells were washed three times in PBS. Cells were stained with crystal violet (0.05%, 15 min on orbital shaker) and dissolved in acetic acid (10%, 15 min on orbital shaker). Absorbance was measured at 590 nm using a spectrophotometer multiwell plate reader.

### Flow cytometry experiments

#### Cell cycle

After 24 h treatment, BT474 cells were collected and fixed in ethanol (70%, cold) for 30 min. Cell pellets were washed in PBS + 2% BSA and incubated in dark, 1 h at 4 °C with Propidium iodide/RNAse staining solution (Immunostep).

#### Cell death and apoptosis

Adherent and floating BT474 cells were collected after 72 h of treatment and stained for 1 h with Annexin Binding Buffer containing Annexin V-DT-634 and Propidium iodide (2 mg/mL) (Immunostep). All analyses were performed on a FACSCanto™ II flow cytometer using the FACS Diva software.

### Protein expression analysis: western blotting

BT474 cells were seeded (750.000 cell/100 mm dish), and 24 h later were treated with MZ1 (100 nM), trastuzumab (10 nM), or MZ1 (100 nM) plus trastuzumab (10 nM). For apoptosis protein panel cells were collected 12, 24 and 48 after treatment. For the evaluation of depending time BRD4 and ERBB2 degradation after treatment cells conditions were collected sequentially: first, the 12 h points; the following, the 24 h points; next, the 48 h points; and finally, the 72 h points. In parallel 72 h and 96 h post-seeding non-treated control were collected.

After each treatment, cells were washed with cold PBS and lysed in cold lysis RIPA buffer containing 1% Protease Inhibitor Cocktail for use with mammalian cell and tissue extracts and 1% Phosphatase Inhibitor Cocktail (Sigma-Aldrich, Saint Louis, MO, USA). Insoluble material was removed by centrifugation 10.000 g for 10 min. The protein concentration was determined using BCA (Bicinchoninic acid) protein assay kit (Thermo Fisher Scientific). To perform the Western-blotting, 40 μg protein was separated on 6–15% sodium dodecyl sulfate polyacrylamide gel electrophoresis (SDS-PAGE) and transferred to polyvinylidene difluoride membranes (Merck millipore, Darmstadt, Germany). Blots were blocked in 1xTris-buffered saline (TBS, 100 mM Tris [pH 7.5], 150 mM NaCl) with 0.05% Tween and 1% BSA for 1 h at room temperature. Then, membranes were incubated with the primary antibodies (1xTBS containing 0.05% Tween, overnight) described in Supplementary Table 1. Anti-phospo tyr was used to detect pERBB2. For immunoprecipitation, equal amounts of protein were incubated with Affi-gel pertuzumab conjugated (Biorad) at 4 °C for at least 2 h. Immune complexes were recovered by a short centrifugation at 17,000 g for at least 30 s, followed by three washes with 1 ml ice-cold lysis buffer. Horseradish peroxidase-coupled secondary antibodies (anti-rabbit (1:5.000) or anti-mouse (1:10.000), Santa Cruz Biotechnology) were used to identified protein-bound primary antibodies (1xTBS containing 0.05% Tween with 5% milk, 30 min, RT). Protein bands were detected using ECL Plus Western Blotting Detection System (GE Healthcare, Buckinghamshire, United Kingdom).

### Immunofluorescence

BT474 grown on glass coverslips were washed and fixed in 2% paraformaldehyde for 30 min at room temperature. Monolayers were rinsed twice with PBS and blocked in PBS containing 0.1% Triton X-100 and 0.2% BSA for 10 min (× 3), and subsequently incubated for 3 h with anti-pH2AX (1:300, Cell Signaling). Coverslips were washed three times with PBS containing 0.2% BSA and incubated with an anti-rabbit Cy3 (1:1000) antibody for 30 min. DAPI (300 nM) was added for 30 s and washed with PBS before mounting. Fluorescence imaging of cells were performed using an epifluorescence inverted microscope (DMIRE-2, Leica). Foci were counted in the nuclei using ImageJ. At least 100 nuclei per condition were analyzed.

### In vivo studies (xenograft mice)

BALB/c nu/nu mice (4–5 weeks old, *n* = 12) mammary fat pads were injected with BT474 cells (5 × 10^6^) in 50% of Matrigel (Sigma-Aldrich). Treatment was initiated when tumors reached a volume of 500 mm^3^. Animals was divided in four group (*n* = 4): non-treated control, trastuzumab, MZ1 and combination. MZ1 was intraperitoneally injected at doses of 10 mg/kg 5 days consecutively per week. Herceptin was intraperitoneally injected at doses of 30 mg/kg once a week. Then, tumours were collected, weighted, and stored at − 80 °C. For biochemical analyses, tumours were minced, washed with PBS, and homogenized with Dispomix (L&M Biotech, Holly Springs, NC, USA) in ice-cold lysis buffer (1.5 ml/100 mg of tumour). This homogenate was centrifuged at 10,000×g for 20 min, and the supernatants were transferred to new tubes. Western blotting was performed as described in Protein expression analysis: Western blotting section.

### Microarray analysis of mRNA

For whole-genome profiling, BT474 cells were seeded (750.000 cells/ 100 mm plate). After 24 h of incubation, cells were treated with drugs (trastuzumab (10 nM), MZ1 (100 nM), or a combination of 10 nM trastuzumab and 100 nM MZ1) for 12 or 24 h. Total RNA was obtained from cells using RNeasy Mini Kit (Qiagen, Hilden, Germany) according to manufacturer’s instruction. After extraction, concentration and purity were determined using a NanoDrop ND-1000 spectrophotometer (Thermo Fisher Scientific, USA).

The RNA integrity was assessed using Agilent 2100 Bioanalyzer (Agilent). Labeling and hybridizations were performed according to protocols from Affymetrix. Briefly, 100 ng of total RNA was amplified and labeled using the WT Plus reagent Kit (Affymetrix) and then hybridized to Clariom S human (Affymetrix). Washing and scanning were performed using GeneChip System of Affymetrix (GeneChip Hybridization Oven 645, GeneChip Fluidics Station 450 and GeneChip Scanner 7G).

Genes with different expression values from the control versus treated groups (12 and 24 h) were obtained. A threshold of FC < -2 for downregulated and FC > 2 for upregulated genes are used (with *p* < 0.01). The list of genes was analyzed using the biological function enrichment analyses tool EnrichR (http://www.amp.pharm.mssm.edu/Enrichr/) to identify functions modified by MZ1 and combination. Genes were classified in those specifically modified after treatments with MZ1 or combination, and those commonly shared. Biological process gene ontologies related to similar function with *p* value< 0.01 were selected and grouped in the same process.

### Prognosis study

KM Plotter Online Tool (http://www.kmplot.com) were used as clinical outcome prediction tool. This publicly available database shows the relationship between gene expression and patient outcome in different breast cancer subtypes. Patients were distributed according to the best cutoff values of the gene expression (lowest p value) into “high” vs. “low”. RFS was defined as the time from diagnosis to the first recurrence. The number of breast cancer patients included in HER2+ status is 252.

### Quantitative reverse-transcription PCR

Total RNA was obtained from cells using RNeasy Mini Kit (Qiagen, Hilden, Germany) according to manufacturer’s instruction. After extraction, concentration and purity were determined using a NanoDrop ND-1000 spectrophotometer (Thermo Fisher Scientific, USA) and, subsequently, 1 μg of total RNA was reversely transcribed using RevertAid H Minus First Strand cDNA syntesis Kit (Thermo Fisher Scientific, USA) in a thermocycler (Bio-Rad) under the following reaction conditions: 65 °C for 5 min, 42 °C for 60 min and 70 °C for 10 min. The cDNAs were then subjected to a real-time PCR analysis using Fast SYBR Green Master Mix in StepOnePlus Real-Time PCR system (Applied Biosystems) according to the manufacturer’s instructions. Primer sequences used were described in Supplementary Table 2. An initial step was performed at 95 °C for 5 min, followed by 40 cycles of 95 °C for 15 s and finished by 60 °C for 1 min. Each sample was analyzed in triplicates and cycle threshold (Ct) values of transcripts was determined using StepOne Software v.2.1. Ct values were calculated using 18Sas reference. Untreated control cells were used as control to calculate the Ct value and to determine the X-fold mRNA expression.

### Statistical analysis

We used unpaired t-test for independent samples assay, together with the F test to consider, or not, equal variances or One-way ANOVA assay with Tukey subtype. The level of significance was considered 95% (* *p* ≤ 0.05; ** *p* ≤ 0.01 and *** *p* ≤ 0.001). Software GraphPad Prism 7.0 were used.

## Results

### BET-PROTACs demonstrate anti-tumoral activity alone and in combination with trastuzumab in HER2+ breast cancer cell lines

**Fig. 1:**
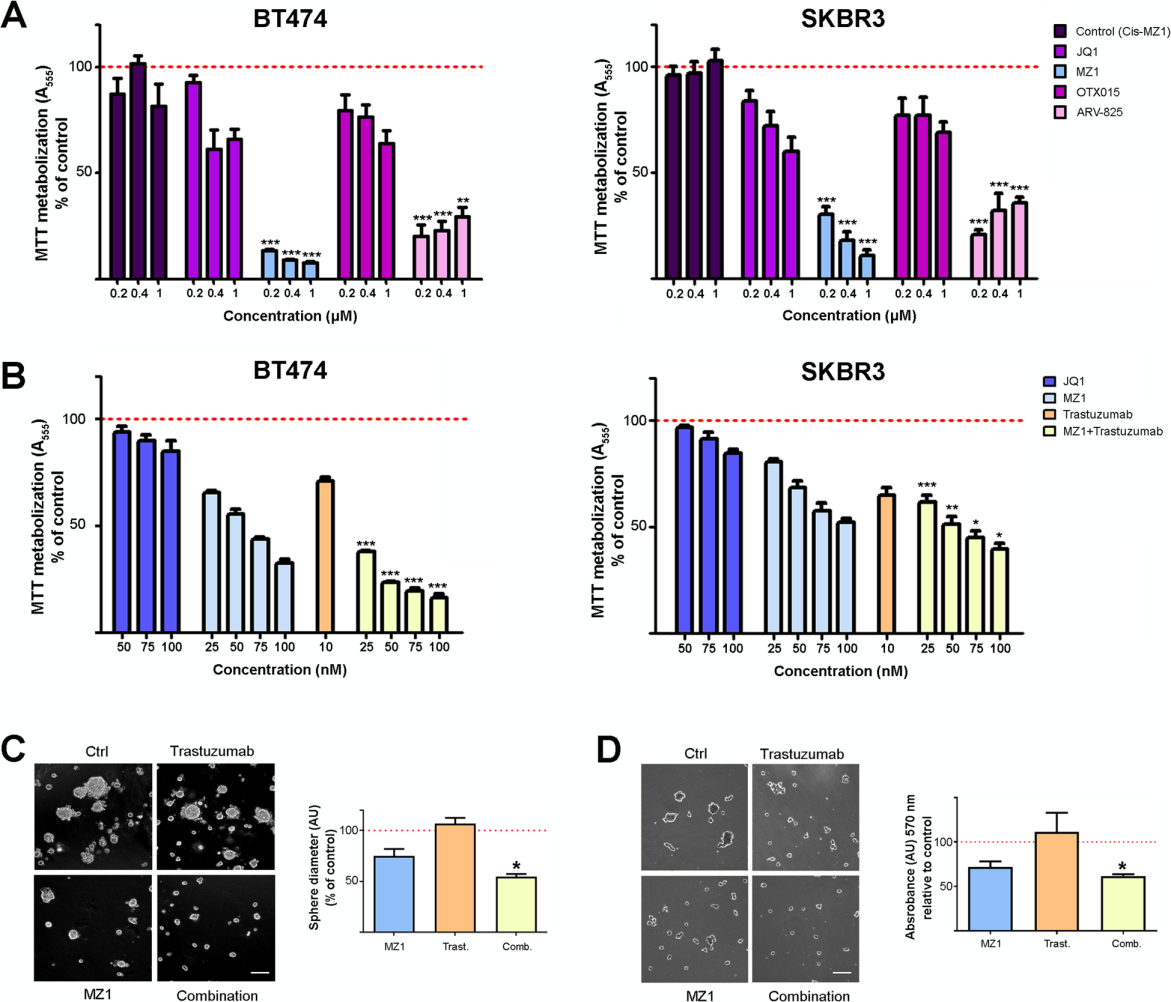
Evaluation of antitumoral activities of MZ1 and the MZ1-Trastuzumab combination in HER2+ breast cancer lines. **a** Cell viability evaluated by MTT assays for BT474 (left) and SKBR3 (right) cells treated with JQ1, OTX015, and their respective PROTACs (MZ1 and ARV-825) at the doses indicated (72 h). Cis-MZ1 was used as control. **b** Cell viability evaluated by MTT assays for BT474 (left) and SKBR3 (right) cells treated with JQ1, MZ1, trastuzumab and the MZ1-trastuzumab combination at the doses indicated (72 h). **c** BT474 cells were seeded in a Matrigel matrix and treated with MZ1 (100 nM), trastuzumab (10 nM) or the MZ1-trastuzumab combination treatments were applied (72 h). Invasion capacity was assessed by measuring sphere diameters of invading 3D structures, and results are presented as percentage referred to untreated cells. Scale bar = 100 μm. **d** Cell adhesion to poly-lysine substrate after 24 h exposure to MZ1 (100 nM), trastuzumab (10 nM) and the MZ1-trastuzumab combination. BT474 cells were treated, trypsinized, and seeded, and percentage of cells adhered to plaque referred to untreated cells after 1 h is presented. Scale bar = 100 μm. *p < 0.05; **p < 0.01; ***p < 0.001

**Fig. 2:**
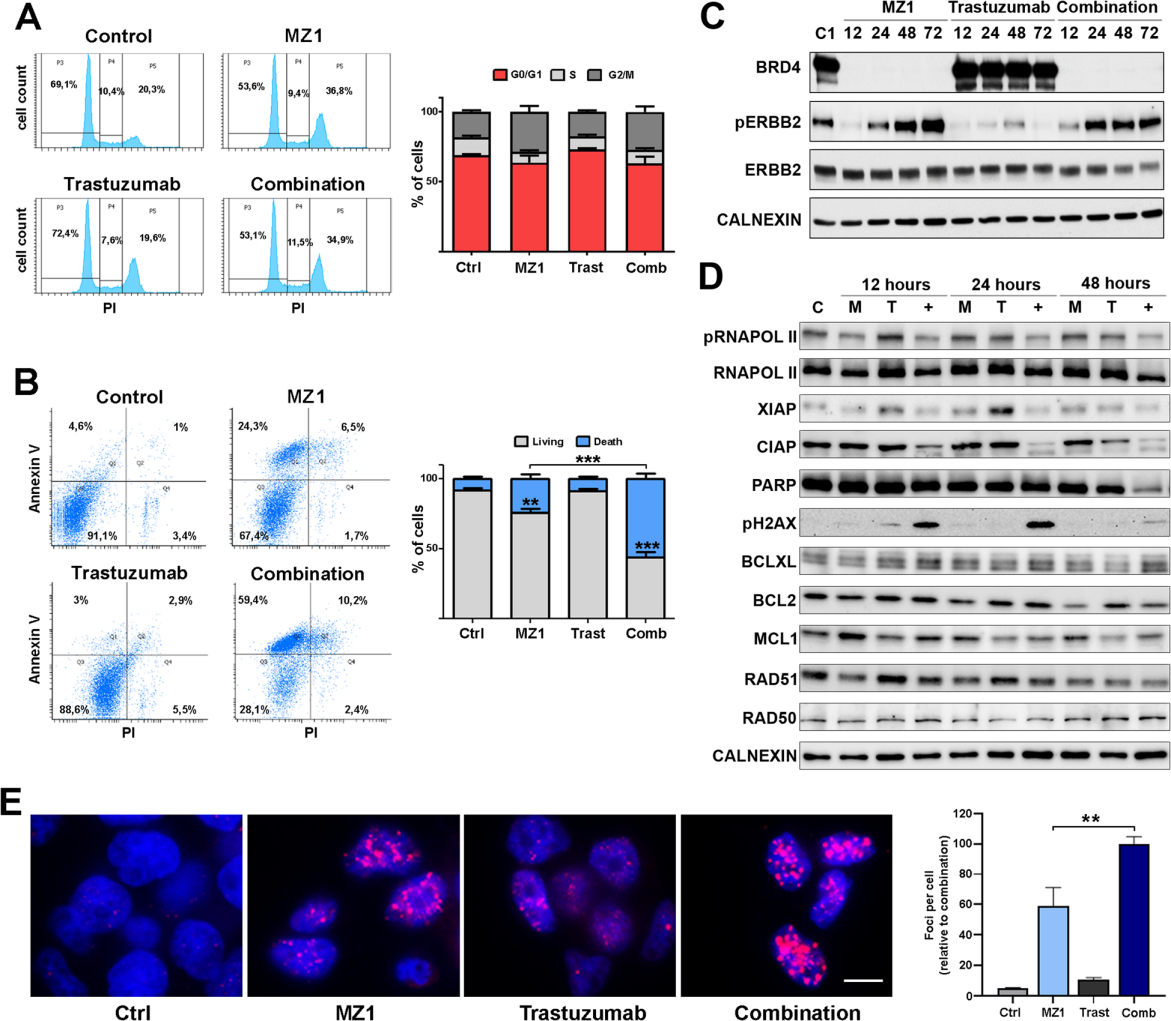
Mechanism of action of MZ1-Trastuzumab combination. **a** BT474 cells were treated with MZ1 (100 nM), trastuzumab (10 nM) or the MZ1-trastuzumab combination for 24 h and cell cycle was evaluated by flow cytometry. Left, flow cytometry plots (Annexin V (AV, y axis) and propidium iodure (PI, x axis). Right, bar graphs show the percentage of cells in G0/G1, S, or G2/M cell cycle phases. **b** BT474 cells were treated with MZ1 (100 nM), trastuzumab (10 nM) or the MZ1-trastuzumab combination for 72 h and cell death was evaluated by flow cytometry with Annexin V (AV) and propidium iodure (PI) staining. Left, flow cytometry plots (Q3, living (AV -, PI -); Q1, early apoptosis (AV +, PI -); Q2, late apoptosis (AV +, PI +); Q4, necrotic (AV-, PI+). Right, bar graphs show the percentage of cells classified in living (AV -, PI -), and death (AV +, PI -, AV +, PI +, AV-, PI+). **c** Western blot showing expression levels of BRD4, pERBB2 and ERBB2 in BT474 cells treated with MZ1, trastuzumab, combination or left untreated at the times indicated. **d** Western blot showing expression level of proteins related to apoptosis and DNA damage in BT474 cells treated with MZ1, trastuzumab, combination or left untreated after 12, 24 and 48 h. **e** Fluorescence images showing pH2AX immunoreactivity (magenta) and DNA staining (blue) were obtained by epifluorescence microscopy after MZ1 (100 nM), trastuzumab (10 nM) or the MZ1-trastuzumab combination treatment in BT474 cells. Bar graphs indicate the number of pH2AX foci per cell quantified using ImageJ. Scale bar = 10 μm. *p < 0.05, **p < 0.01, ***p < 0.001

To analyze the effect of BET-PROTACs on HER2+ breast cancer, we first evaluated the efficacy of two well-known BRD4 degraders, MZ1 (based on the BETi JQ1) and ARV-825 (based on the BETi OTX015) in the HER2-overexpressing breast cancer cell lines BT474 and SKBR3. A significant decrease in cell proliferation measured as MTT metabolization was induced by both compounds compared to their respective BETi, JQ1 and OTX015 (Fig. 1a). MZ1, which is the BET-PROTAC better characterized [9], showed the highest antiproliferative effect. Once their activity was confirmed, we decided to explore the effect of the combination of MZ1 and Trastuzumab. The combination induced a more profound antiproliferative effect than the administration of single agents in both BT474 and SKBR3 cells (Fig. 1b). A synergistic interaction between MZ1 and Trastuzumab in BT474 cells and an additive interaction in SKBR3 cells was observed (Supp.Fig. 1). However, this synergistic effect was not observed in a normal breast epithelial cell line, which does not express ERBB2 (MCF10A) (Supp.Fig. 2). We next evaluated in BT474 cells the effect of these compounds on additional oncogenic properties such as invasion and cell adhesion, analyzing the sphere diameters of invading 3D structures and the number of cells reseeded to fibronectin coated plaques, respectively. The MZ1-trastuzumab combination demonstrated to be the most effective treatment against both biological functions (Fig. 1c and d).

### MZ1-trastuzumab combination induces apoptosis

To explore if the effect observed on proliferation was due to cell cycle arrest or induction of apoptosis, flow cytometry experiments were performed in BT474 cells. No clear modifications were identified in any phase of the cell cycle upon administration of the drugs, although a slight augment in G2/M was observed (Fig. 2a). However, although no effect was found in response to the individual treatment with trastuzumab, a significant increase in cell death was observed upon MZ1 and, more drastically, following exposure to the combination (Fig. 2b). In fact, the combination led to double the number of apoptotic cells referred to MZ1 individual treatment (24% vs 57%).

### Mechanism of action of the combination includes DNA damage and deregulation of the apoptosis machinery

To confirm the effect of the drugs on their direct targets, BRD4 and ERBB2 levels were examined after treatment with MZ1, trastuzumab or the combination. MZ1 efficiently degraded BRD4 as early as 12 h. In the case of ERBB2, a minor time-dependent downregulation of this protein was identified only in the case of the combination (Fig. 2c). At short time points, all treatments efficiently reduced the amount of phosphorylated ERBB2. Conversely, although this effect was maintained in the case of trastuzumab, an increase in pERBB2 was observed in cells treated with MZ1 and the MZ1-trastuzumab combination after a longer time exposure.

**Fig. 3:**
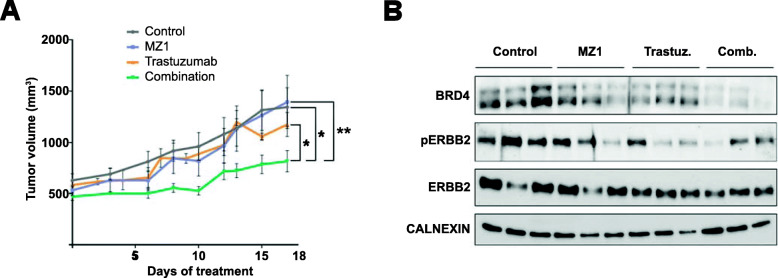
In vivo analysis of xenografted tumors treated with MZ1, trastuzumab or MZ1-trastuzumab. **a** Graphical representation of the tumor volumes (mm^3^) of BT474-derived tumors treated with either MZ1 (10 mg/kg), trastuzumab (30 mg/Kg) or MZ1-trastuzumab combination along the days of treatment. Mean of tumor volume ± SEM is indicated. **b** Western blot showing the expression levels of BRD4, pERBB2 and ERBB2 in tumors treated with MZ1, trastuzumab, combination or left untreated. *p < 0.05; **p < 0.01 (t-test one-tail, unpaired)

Next, a biochemical assessment of the apoptotic machinery components showed a reduction in the expression of proteins involved in apoptosis, such as those of the IAPs (Inhibition Apoptosis Proteins) family, like cIAP-1 and XIAP (Fig. 2d). The reduction of PARP levels, along with increases in H2AX phosphorylation and pH2AX foci per cell (Fig. 2d-e), confirmed the presence of this effect. Of note, the induction of DNA damage measured by pH2AX was observed by Western blot and immunofluorescence analysis, as early as 6 h after treatment with the MZ1-trastuzumab combination (Supp.Fig. 3). No significant changes were observed among other proteins evaluated. This set of data demonstrated an effect on proteins involved in both apoptosis and DNA damage after treatment with the drug combination.

### Antiproliferative activity of MZ1-trastuzumab combination in vivo

**Fig. 4:**
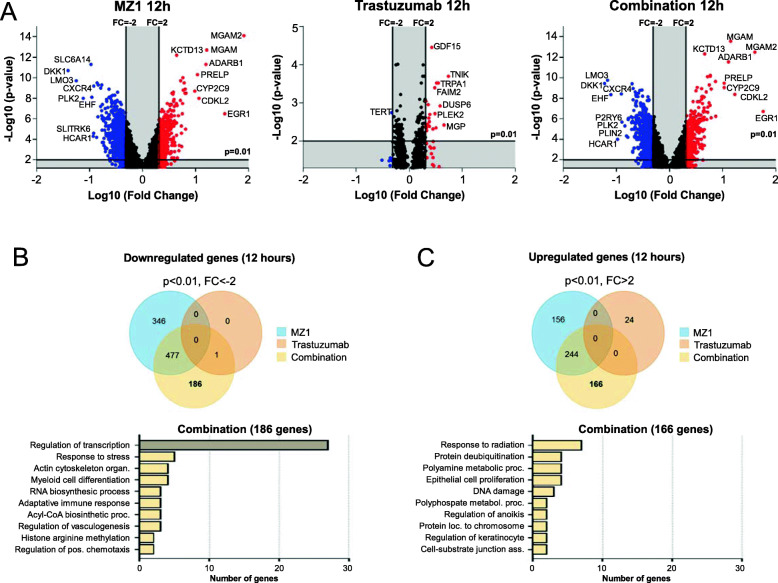
Analysis of a transcriptomic array in cells treated with MZ1, trastuzumab or MZ1-trastuzumab (12 h). **a** Volcano plots (*p*-value (−log 10) vs Fold Change (log10)) of BT474 cells gene expression profile after 12 h treatment with MZ1, trastuzumab, and combination. Upregulated genes (FC > 2) are show in red and downregulated genes (FC < -2) are show in blue. **b** Venn diagram showing the number of downregulated genes in each treatment condition using p-value< 0.01 and FC < -2 as threshold. Bottom bar graph, functional analyses of the 186 altered genes after combination treatment using EnrichR Online Tool. Biological process gene ontologies (*p* < 0.01) grouped are shown. **c** Venn diagram showing the number of upregulated genes in each treatment condition using p-value < 0.01 and FC < -2 as threshold. Bottom bar graph, functional analyses of the 166 altered genes after combination treatment using EnrichR Online Tool. Biological process gene ontologies (*p* < 0.01) grouped are shown

To explore the antitumoral action of the MZ1-trastuzumab combination in an in vivo model, BT474 cell lines were xenografted in mice. Mice were randomized in 4 groups (non-treated, treated with MZ1, Trastuzumab, or the MZ1-Trastuzumab combination). As can be observed in Fig. 3a, only the administration of MZ1 and trastuzumab induced a reduction in tumor progression, while individual treatments failed to do so. Western blots were performed to evaluate the expression of BRD4 and ERBB2 in tumor lysates at the end of treatment. Average expression of BRD4 in tumors was lower in MZ1-trastuzumab treated mice compared with non-treated tumors and single therapies, with no clear modifications of ERBB2 levels (Fig. 3b and suppl.Fig. 4).

### Transcriptomic analysis identifies deregulation of genomic programs

**Fig. 5:**
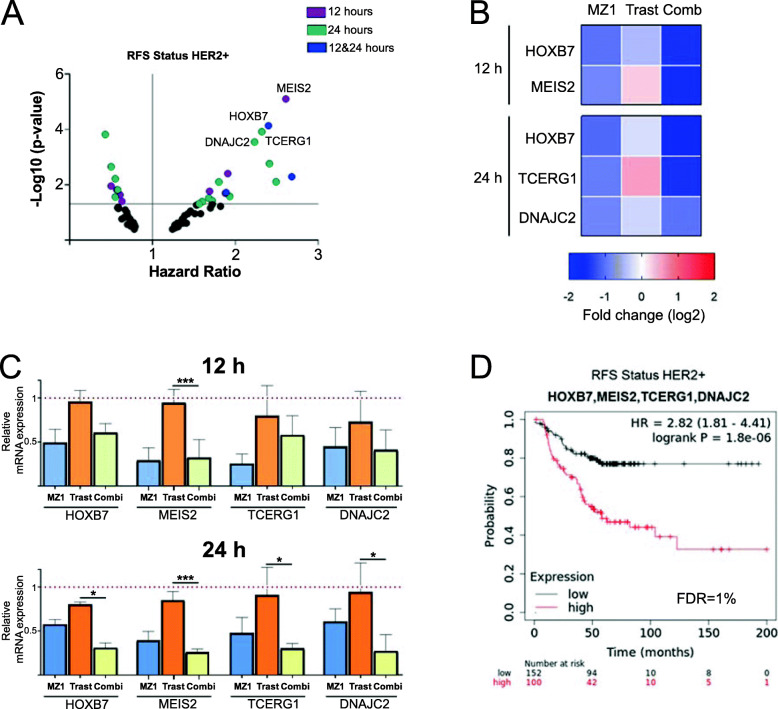
Identification of transcription factors downregulated with the combination. **a** *p* Value vs Hazard Ratio graph indicating transcription factors altered after combination treatment at 12, 24 h or both. Relapse Free Survival (RFS) for HER2^+^ status on the online tool Kaplan-Meier plotter was applied. Genes with no association prognosis value (*p* > 0.05) are shown in black. **b** Expression level heat map of the transcription factors identified (HOXB7, MEIS2, TCERG1, DNAJC2) after 12 and/or 24 h of treatment with MZ1, trastuzumab, and combination. **c**, mRNA expression levels of transcription factor identified genes after 12 or 24 h of treatment with the MZ1, trastuzumab, and combination. *p < 0.05; **p < 0.01; ***p < 0.005. **d** Kaplan-Meier survival plots of the combined selected genes (HOXB7, MEIS2, TCERG1, DNAJC2) signature expression level and patient prognosis in RFS and HER2+ status conditions

Next, we sought to study the transcriptomic profile implicated in the activity of MZ1, trastuzumab, and the MZ1-trastuzumab combination in HER2+ cells. To do so, a microarray expression analysis was performed in BT474 cells at 12 (Fig. 4) and 24 h (Supp.Fig. 5). While trastuzumab barely affected gene expression, both MZ1 and MZ1-Trastuzumab down- and upregulated a substantial number of transcripts, being most of them commonly shared in both treatment groups (Fig. 4a-c). Indeed, focusing on genes specifically modified after the combination treatment, a functional analysis was performed to classify the identified genes into Gene Ontology biological processes. A clear enrichment in the “regulation of transcription” function was observed among downregulated genes (Fig. 4b, bottom). Conversely, we did not find a majority function among upregulated genes (Fig. 4c, bottom). The same analysis was performed at 24 h observing similar findings (Supp.Fig. 5). Interestingly, at that time point an increase in genes participating in metabolic processes was also observed. Among the different treatment scenarios, genes classified with Gene Ontology biological processes (Venn diagrams) are shown in Supp.Fig. 6. In addition, we aimed to compared the transcriptomic signature obtained with our compounds with previously published in relation to resistance to chemotherapy, as performed by other groups [22]. A gene drug resistance signature (NCBI, Accession: GPL1124) was compared with the down- and upregulated transcripts that we obtained after the administration of the different treatments (FC < -2, FC > 2, Supp.Fig. 7). A limited number of genes from the drug resistance signature were commonly shared what demonstrates that the mechanism of action of our combinations is different from previously described.

Then, we sought to analyze the specific genes included in the principal downregulated function, “regulation of transcription”. First, we explored the association of the identified genes included in this function with clinical outcome. Among all the genes investigated, we found four genes, HOXB7, MEIS2, TCERG1, and DNAJC2, as significantly associated with detrimental prognosis in patients, suggesting that the expression of these genes might have an oncogenic role in HER2+ tumors (Fig. 5a and Supp.Table 3). A heatmap of the expression levels of the four identified genes following the different treatments shows the marked decrease in the expression of the four genes after treatment with MZ1 that was observed in the transcriptomic analysis, which was even higher when the PROTAC was combined with Trastuzumab (Fig. 5b). These results were confirmed through a qPCR analysis. As can be seen, treatment with both MZ1 and the combination led to the downregulation of HOXB7, MEIS2, TCERG1, and DNAJC2, being this effect even more prominent with the combination (Fig. 5c). Of note DNAJC2 was similarly affected by MZ1 alone or when combined. Finally, we explored the association of this four-genes signature with prognosis. The combined analysis of the four genes revealed a higher predicted potential compared with individual genes to identify patients with a detrimental outcome (HR 2.82 CI 1.81–4.41, *p* = 1.8e-06) (Fig. 5d). These results indicate the oncogenic role of these genes in HER2 + tumors and the clear effect on them by the treatment with the combination.

## Discussion

Identification of druggable vulnerabilities to augment the efficacy of approved therapeutics is an excellent manner to improve cancer therapy. For HER2+ breast cancer tumors, trastuzumab is the standard of care treatment used when combined with chemotherapeutics [23]. Unfortunately, not all patients respond to these combinations, and most of them progress within a specific period of time [2, 3]. Hence, the discovery of novel trastuzumab-based combinations that improve efficacy in preclinical models is a strategy to pursue. In this regard, PROTACs constitute a new family of compounds that have shown relevant preclinical activity by promoting the degradation of oncogenic proteins [8]. In this work, we focused on PROTACs based on BETi, as they constitute a novel strategy especially considering previous data suggesting that expression of BRDs is involved in detrimental outcome in HER2+ patients [21]. Of note, in the case of ARV-825, we observe a decrease of its antiproliferative effect when drug concentration increases. As previously demonstrated, PROTACs are subjected to a “hook effect” due to ineffective binary complexes formation [24]. As MZ1 mitigates the hook effect due to its positive cooperativity with respect to the assembly of ternary complexes [9], we decided to explore the combination of MZ1 and trastuzumab in preclinical models of HER2+ breast cancer.

We first observed a clear anti-proliferative activity of MZ1 in HER2 overexpressing cells, suggesting that MZ1 is an active agent in this breast cancer subtype. Indeed, this effect augmented when administered in combination with trastuzumab just in cancer cells and not in a non-tumorigenic breast cell line. The unique synergistic/additive interaction between MZ1 and trastuzumab in the HER2+ cell line suggests a higher therapeutic index.

Biochemical studies demonstrated that MZ1 efficiently reduced the expression of BRD4, and all the treatments reduced the amount of phosphorylated ERBB2 at short time exposure. The increase in pERBB2 observed at longer times in the presence of MZ1 and the combination could be secondary to a compensatory effect mediated by the increase in the activity of phosphatases. Overall, our findings reinforce previous studies describing that the inhibition of both BRD4 and the ERBB2 downstream PI3K pathway produces antitumoral activity in different tumor cell lines [25].

Triggering apoptosis has been described as a vulnerability to augment the efficacy of anti-HER2 therapies in HER2+ tumors [19]. Our results showed that MZ1 was able to induce apoptosis, an effect that is enhanced when combined with trastuzumab. To elucidate the mechanism of action, biochemical studies were performed. Western blot analysis identified a clear deregulation of components of both apoptosis and DNA damage machinery. Decreased expression of RNA polymerase II after MZ1 plus trastuzumab treatment could be responsible for the diminished XIAP and c-IAP-1, proteins activated in order to repair DNA damage [26], resulting finally in a large increase in H2AX phosphorylation, the main determinant of the presence of damage in the double strand DNA [27].

To validate the effect observed in cellular models, we explored the efficacy in an orthotopic in vivo model. BT474 cells were xenografted in the mammary gland of nude mice and then treated with each agent alone or the combination. The reduction of tumor growth of both agents given in combination showed a higher efficacy than single agent administration, with no toxicity concerns, what supports the potential use of the combination in vivo. Biochemical analysis of these tumors confirmed the reduction of BRD4 observed in cell lines, while again HER2 levels showed no clear changes.

A transcriptomic expression microarray analysis performed after administration of the different treatments allowed us to get further insights into the mechanism of action of the combination. Both MZ1 alone and the combination downregulated a great number of genes. As anticipated, having in mind the subsequent effect mediated by the degradation of BRD4 [28], most of them were modulators of transcription.

Detailed evaluation of the transcription factors downregulated by the MZ1-trastuzumab combination led us to the identification of four genes: HOXB7, MEIS2, TCERG1, and DNAJC2, which were associated with detrimental patient prognosis when expressed in HER2+ patients. HOXB7 is a transcription factor previously related to poor outcome in breast cancer, affecting epidermal to mesenchymal transition or angiogenesis (reviewed in [29]). Interestingly, HOXB7 plays a role in DNA repair in addition to its function as transcriptional regulator [30]. MEIS2, which belongs to the same family of HOXB7, has been also reported to be a master regulator in breast cancer [31]. On the other hand, TCERG1 is involved in alternative splicing inhibiting the elongation of transcripts from target promoters by binding to RNA polymerase II, and has been identified as a regulator of proapoptotic proteins [32]. Finally, DNAJC2 has been described as a chromatin associated transcription factor linked to different types of cancer [33]. The association of the four transcription factors with detrimental outcome, alone or when used as a combined signature, provided evidence of their potential oncogenic role in patients. As MZ1-trastuzumab efficiently reduced the expression of all these genes, we hypothesize that this gene panel could be used to identify patients willing to respond to the drug combination.

## Conclusions

We report the synergistic/additive interaction between the anti-HER2 monoclonal antibody trastuzumab and the BET-PROTAC MZ1 in HER2+ breast tumors, through the downregulation of transcriptional genes, cell proliferation inhibition, and the modulation of apoptotic and DNA damage pathways. These findings pave the way for future clinical studies in patients.

## Supplementary Information

**Additional file 1: Figure S1.** A, Dose response MTT assays for evaluated MZ1 and Trastuzumab effect in BT474 and SKBR3 cells. B, BT474 and SKBR3 cells were incubated with increasing concentrations of MZ1 alone or in combination with increasing concentrations of Trastuzumab at indicated doses for 72 h. Cell viability was assessed by MTT assay. C, Quantitation of synergistic anti-proliferative effect of MZ1 and trastuzumab in BT474 and SKBR3 cells. Combination indexes (CI) for the different drug combinations were obtained using CalcuSyn program (t-test one-tail, unpaired) *p < 0.05; **p < 0.01; ***p < 0.001.

**Additional file 2: Figure S2.** MCF10A cells were incubated with increasing concentrations of MZ1 alone or in combination with increasing concentrations of Trastuzumab at indicated doses for 72 h. An unpaired t-test one-tail was used to evaluate statistical significance. *ns*, not significant.

**Additional file 3: Figure S3.** A, Western blot showing expression level in BT474 cells of pH2AX in BT474 cells treated after 3, 6 and 12 h with MZ1 (100 nM), trastuzumab (10 nM), or the MZ1-trastuzumab combination, or left untreated. B, Fluorescence images of BT474 cells showing pH2AX immunoreactivity (magenta) and DNA staining (blue) obtained by epifluorescence microscopy after MZ1 (100 nM), trastuzumab (10 nM) or the MZ1-trastuzumab combination treatments at the same time points. Scale bar = 10 μm.

**Additional file 4: Figure S4.** Quantification of the bands detected in Western blot analysis from Fig. 3b. Densitometry was measured with ImageJ software. BRD4, pERBB2 and ERBB2 bands were normalized to their respective loading controls. Bar graphs are referred to the non-treated samples. *p < 0.05.

**Additional file 5: Figure S5.** Analysis of a transcriptomic array in cells treated with MZ1, Trastuzumab or MZ1-Trastuzumab (24 h). A, Volcano plots (*p*-value (−log 10) vs Fold Change (log10)) of BT474 cells gene expression profile after 24 h treatment with MZ1, trastuzumab, and combination. Upregulated genes (FC > 2) are show in red and downregulated genes (FC < -2) are show in blue. B, Venn diagram showing the number of downregulated genes in each treatment using *p*-value< 0.01 and FC < -2 as threshold. Bottom bar graph, functional analyses of the 401 altered genes after combination treatment using EnrichR Online Tool. Biological process gene ontologies (*p* < 0.01) grouped are shown. C, Venn diagram showing the number of upregulated genes in each treatment condition using *p*-value < 0.01 and FC < -2 as threshold. Bottom bar graph, functional analyses of the 266 altered genes after combination treatment using EnrichR Online Tool. Biological process gene ontologies (*p* < 0.01) grouped are shown.

**Additional file 6: Figure S6.** Venn diagram showing the number of downregulated or upregulated genes (A, 12 h and B, 24 h) in MZ1 and combination treatment condition. Functional analyses of the altered genes after each treatment using EnrichR Online Tool are shown. Biological process gene ontologies (*p* < 0.01) grouped are shown. Genes were classified in those specifically modified after treatments with MZ1 or combination, and those commonly shared. Results as presented as percentage of total genes of each biological process.

**Additional file 7: Figure S7.** Venn diagram showing the number of downregulated or upregulated genes (A, 12 h and B, 24 h) in each treatment condition and a drug resistance gene list (DR) (NCBI, Accession: GPL1124). DR and each condition overlapping genes are specified.

**Additional file 8: Table S1.** List of primary human monoclonal/polyclonal antibodies. **Table S2.** Primer sequences used for HOXB7, MEIS2, TCERG1, and, DNAJC2 genes qPCR amplification. **Table S3.** Genes selection related to regulation of transcription process. Prognosis value of regulation of transcription genes altered in combination for 12 h, 24 h, or both. Selected genes using a threshold of HR > 1, *p*-value< 0.05, and FDR-value< 20% are highlighted in grey.

## Data Availability

The data that support the findings of this study are available from the corresponding authors upon reasonable request.

## Abbreviations

BCA: Bicinchoninic acid
BET: Bromodomain extra-terminal
BETi: Bromo and extraterminal domain inhibitors
BSA: Bovine serum albumin
DMEM: Dulbecco’s modified eagle’s medium
DMSO: Dimetilsulfoxide
DNA: deoxyribonucleic acid
HER2: Human epidermal growth factor receptor 2
MTT: Thiazolyl blue tetrazolium bromide
PBS: Phosphate buffer saline
PROTAC: Proteolysis targeting chimera
RIPA: Radioimmunoprecipitation assay
RNA: Ribonucleic acid
T-DM1: Antibody-drug conjugate trastuzumab-emtansine

## References

[1] Slamon DJ, Godolphin W, Jones LA, Holt JA, Wong SG, Keith DE, Levin W, Stuart S, Udove J, Ullrich A (1989). Studies of the HER-2/neu proto-oncogene in human breast and ovarian cancer. Science..

[2] Ocaña A, Pandiella A (2013). Targeting HER receptors in cancer. Curr Pharm Des.

[3] García-Alonso S, Ocaña A, Pandiella A (2020). Trastuzumab emtansine: mechanisms of action and resistance, clinical progress, and beyond. Trends Cancer.

[4] Shu S, Polyak K (2016). BET bromodomain proteins as cancer therapeutic targets. Cold Spring Harb Symp Quant Biol.

[5] Khandekar D, Tiriveedhi V. Role of BET inhibitors in triple negative breast cancers. Cancers. 2020;12(4). 10.3390/cancers12040784. 10.3390/cancers12040784 PMC7226117 32218352

[6] Ocaña A, Nieto-Jiménez C, Pandiella A (2017). BET inhibitors as novel therapeutic agents in breast cancer. Oncotarget.

[7] Pérez-Salvia M, Esteller M (2017). Bromodomain inhibitors and cancer therapy: from structures to applications. Epigenetics.

[8] Khan S, He Y, Zhang X, Yuan Y, Pu S, Kong Q, Zheng G, Zhou D (2020). PROteolysis TArgeting chimeras (PROTACs) as emerging anticancer therapeutics. Oncogene..

[9] Ocaña A, Pandiella A (2020). Proteolysis targeting chimeras (PROTACs) in cancer therapy. J Exp Clin Cancer Res CR.

[10] Zengerle M, Chan K-H, Ciulli A (2015). Selective small molecule induced degradation of the BET bromodomain protein BRD4. ACS Chem Biol.

[11] Sun B, Fiskus W, Qian Y, Rajapakshe K, Raina K, Coleman KG, Crew AP, Shen A, Saenz DT, Mill CP, Nowak AJ, Jain N, Zhang L, Wang M, Khoury JD, Coarfa C, Crews CM, Bhalla KN (2018). BET protein proteolysis targeting chimera (PROTAC) exerts potent lethal activity against mantle cell lymphoma cells. Leukemia..

[12] Noblejas-López MDM, Nieto-Jimenez C, Burgos M, Gómez-Juárez M, Montero JC, Esparís-Ogando A (2019). Activity of BET-proteolysis targeting chimeric (PROTAC) compounds in triple negative breast cancer. J Exp Clin Cancer Res CR.

[13] Corrales-Sánchez V, Noblejas-López MDM, Nieto-Jiménez C, Pérez-Peña J, Montero JC, Burgos M (2020). Pharmacological screening and transcriptomic functional analyses identify a synergistic interaction between dasatinib and olaparib in triple-negative breast cancer. J Cell Mol Med.

[14] Alcaraz-Sanabria A, Nieto-Jiménez C, Corrales-Sánchez V, Serrano-Oviedo L, Andrés-Pretel F, Montero JC, Burgos M, Llopis J, Galán-Moya EM, Pandiella A, Ocaña A (2017). Synthetic lethality interaction between Aurora kinases and CHEK1 inhibitors in ovarian cancer. Mol Cancer Ther.

[15] Gandullo-Sánchez L, Capone E, Ocaña A, Iacobelli S, Sala G, Pandiella A (2020). HER3 targeting with an antibody-drug conjugate bypasses resistance to anti-HER2 therapies. EMBO Mol Med.

[16] Pertuzumab and Trastuzumab: the rationale way to synergy - PubMed. [cited 2020 Jul 3]. Available from: https://pubmed.ncbi.nlm.nih.gov/27275646/

[17] Segovia-Mendoza M, González-González ME, Barrera D, Díaz L, García-Becerra R (2015). Efficacy and mechanism of action of the tyrosine kinase inhibitors gefitinib, lapatinib and neratinib in the treatment of HER2-positive breast cancer: preclinical and clinical evidence. Am J Cancer Res.

[18] Gianni L, Eiermann W, Semiglazov V, Manikhas A, Lluch A, Tjulandin S (2010). Neoadjuvant chemotherapy with trastuzumab followed by adjuvant trastuzumab versus neoadjuvant chemotherapy alone, in patients with HER2-positive locally advanced breast cancer (the NOAH trial): a randomised controlled superiority trial with a parallel HER2-negative cohort. Lancet Lond Engl.

[19] Díaz-Rodríguez E, Pérez-Peña J, Ríos-Luci C, Arribas J, Ocaña A, Pandiella A (2019). TRAIL receptor activation overcomes resistance to trastuzumab in HER2 positive breast cancer cells. Cancer Lett.

[20] Ocana A, Gil-Martin M, Antolín S, Atienza M, Montaño Á, Ribelles N, Urruticoechea A, Falcón A, Pernas S, Orlando J, Montero JC, Escudero MJ, Benito S, Caballero R, Carrasco E, Rojo F, Pandiella A, Ruiz-Borrego M (2019). Efficacy and safety of dasatinib with trastuzumab and paclitaxel in first line HER2-positive metastatic breast cancer: results from the phase II GEICAM/2010-04 study. Breast Cancer Res Treat.

[21] Pérez-Pena J, Páez R, Nieto-Jiménez C, Sánchez VC, Galan-Moya EM, Pandiella A, et al. Mapping Bromodomains in breast cancer and association with clinical outcome. Sci Rep. 2019;9 [cited 2020 Jul 21]. Available from: https://www.ncbi.nlm.nih.gov/pmc/articles/PMC6450889/. 10.1038/s41598-019-41934-3 PMC6450889 30952871

[22] Ciocan-Cartita CA, Jurj A, Zanoaga O, Cojocneanu R, Pop L-A, Moldovan A, Moldovan C, Zimta AA, Raduly L, Pop-Bica C, Buse M, Budisan L, Virag P, Irimie A, Gomes Dias SM, Berindan-Neagoe I, Braicu C (2020). New insights in gene expression alteration as effect of doxorubicin drug resistance in triple negative breast cancer cells. J Exp Clin Cancer Res CR.

[23] Kreutzfeldt J, Rozeboom B, Dey N, De P (2020). The trastuzumab era: current and upcoming targeted HER2+ breast cancer therapies. Am J Cancer Res.

[24] Hughes SJ, Ciulli A (2017). Molecular recognition of ternary complexes: a new dimension in the structure-guided design of chemical degraders. Essays Biochem.

[25] Stratikopoulos EE, Dendy M, Szabolcs M, Khaykin AJ, Lefebvre C, Zhou M-M, Parsons R (2015). Kinase and BET inhibitors together clamp inhibition of PI3K signaling and overcome resistance to therapy. Cancer Cell.

[26] Marivin A, Berthelet J, Plenchette S, Dubrez L (2012). The Inhibitor of Apoptosis (IAPs) in adaptive response to cellular stress. Cells.

[27] Rothkamm K, Barnard S, Moquet J, Ellender M, Rana Z, Burdak-Rothkamm S (2015). DNA damage foci: meaning and significance. Environ Mol Mutagen.

[28] Shi J, Vakoc CR (2014). The mechanisms behind the therapeutic activity of BET bromodomain inhibition. Mol Cell.

[29] Errico MC, Jin K, Sukumar S, Carè A (2016). The widening sphere of influence of HOXB7 in solid tumors. Cancer Res.

[30] Rubin E, Wu X, Zhu T, Cheung JCY, Chen H, Lorincz A, Pandita RK, Sharma GG, Ha HC, Gasson J, Hanakahi LA, Pandita TK, Sukumar S (2007). A role for the HOXB7 homeodomain protein in DNA repair. Cancer Res.

[31] Tapia-Carrillo D, Tovar H, Velazquez-Caldelas TE, Hernandez-Lemus E (2019). Master regulators of signaling pathways: an application to the analysis of gene regulation in breast cancer. Front Genet.

[32] Montes M, Cloutier A, Sánchez-Hernández N, Michelle L, Lemieux B, Blanchette M (2012). TCERG1 regulates alternative splicing of the Bcl-x gene by modulating the rate of RNA polymerase II transcription. Mol Cell Biol.

[33] Aloia L, Demajo S, Croce LD (2015). ZRF1: a novel epigenetic regulator of stem cell identity and cancer. Cell Cycle.

