# Supplementary figures and images for: MZ1 co-operates with trastuzumab in HER2 positive breast cancer
Source: J Exp Clin Cancer Res. 2021 Mar 19;40:106. doi: 10.1186/s13046-021-01907-9 (PMC7980639; doi:10.1186/s13046-021-01907-9)

A

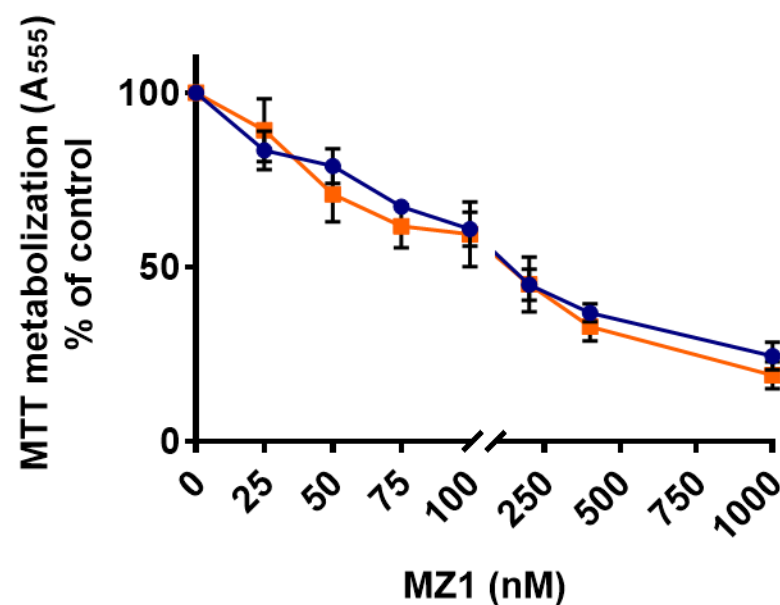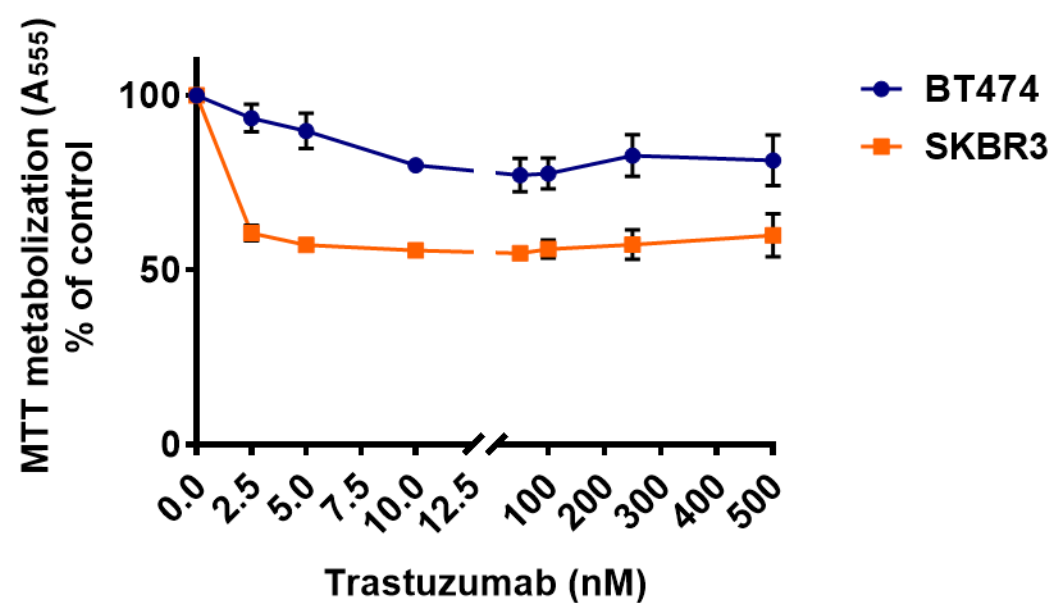

B

BT474

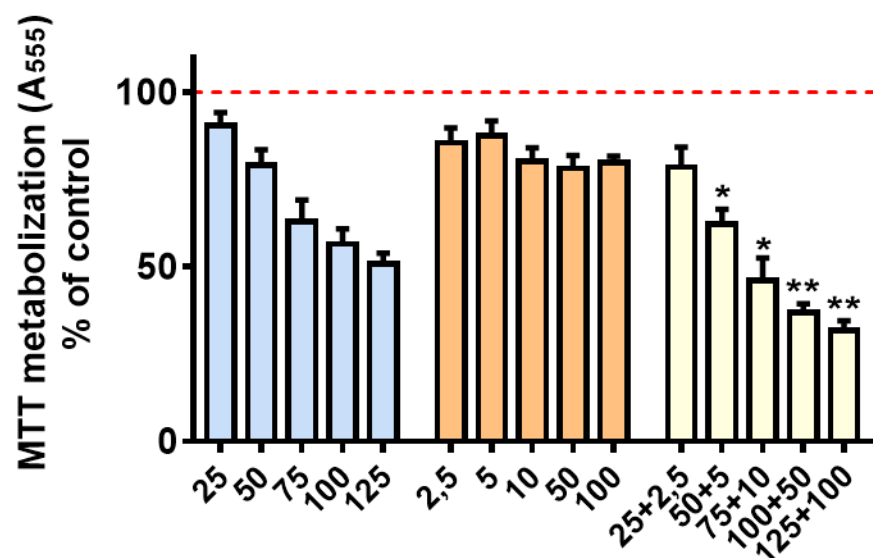

SKBR3

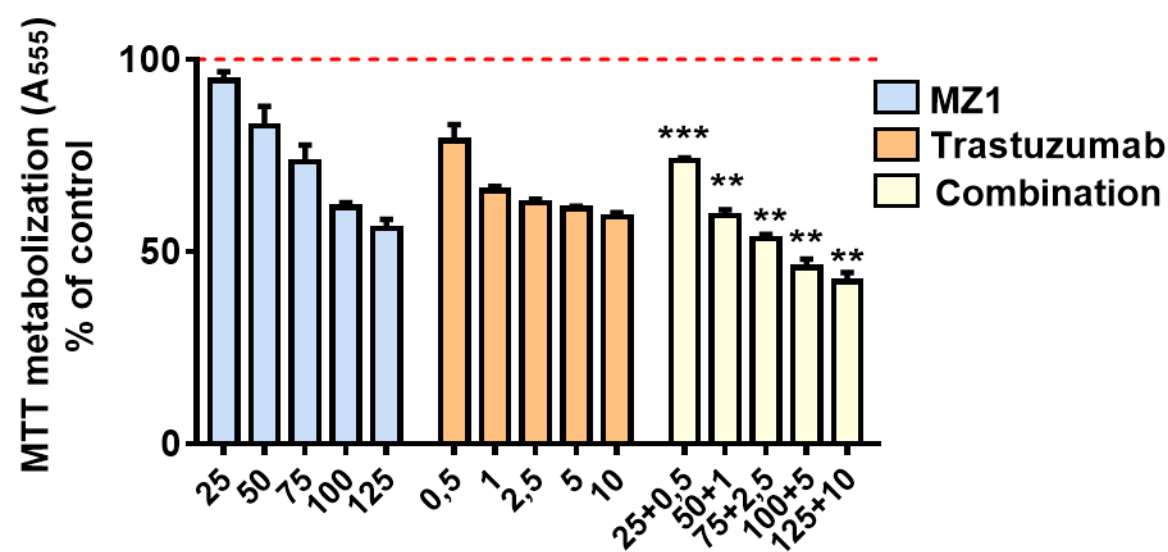

C

BT474

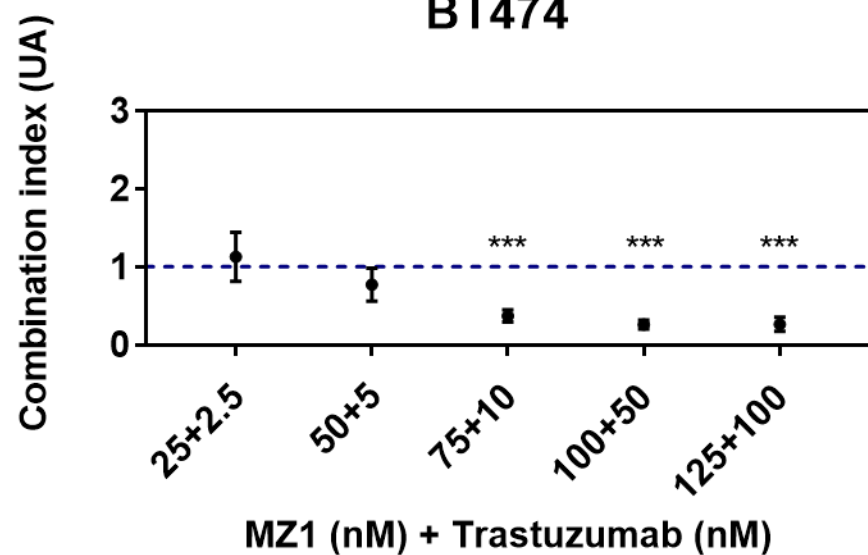

SKBR3

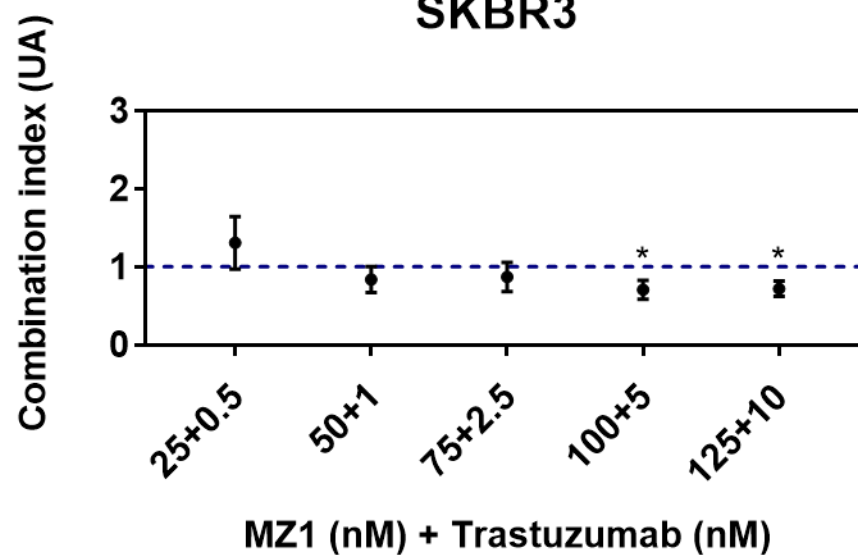

Supplement: Supplementary file 1 — Additional file 1: Figure S1. A, Dose response MTT assays for evaluated MZ1 and Trastuzumab effect in BT474 and SKBR3 cells. B, BT474 and SKBR3 cells were incubated with increasing concentrations of MZ1 alone or in combination with increasing concentrations of Trastuzumab at indicated doses for 72 h. Cell viability was assessed by MTT assay. C, Quantitation of synergistic anti-proliferative effect of MZ1 and trastuzumab in BT474 and SKBR3 cells. Combination indexes (CI) for the different drug combinations were obtained using CalcuSyn program (t-test one-tail, unpaired) *p < 0.05; **p < 0.01; ***p < 0.001. [file 13046_2021_1907_MOESM1_ESM.pdf]

## MCF10A

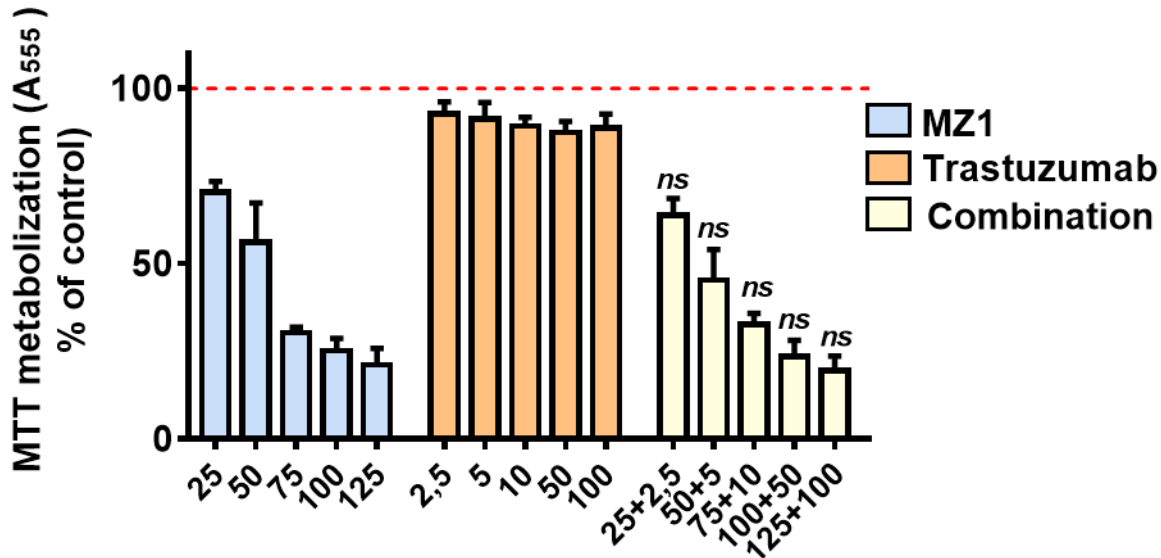

Supplement: Supplementary file 2 — Additional file 2: Figure S2. MCF10A cells were incubated with increasing concentrations of MZ1 alone or in combination with increasing concentrations of Trastuzumab at indicated doses for 72 h. An unpaired t-test one-tail was used to evaluate statistical significance. ns, not significant. [file 13046_2021_1907_MOESM2_ESM.pdf]

**A**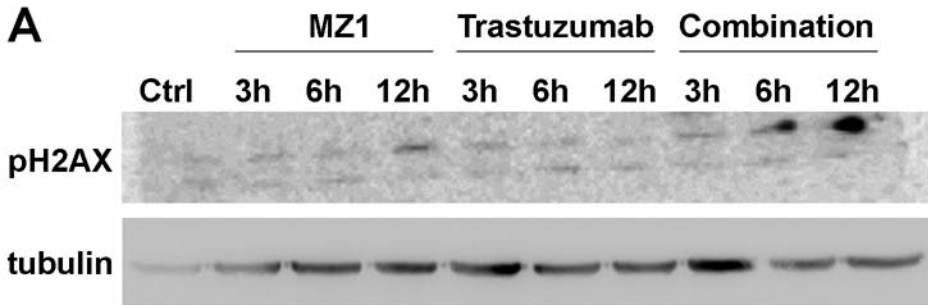**B**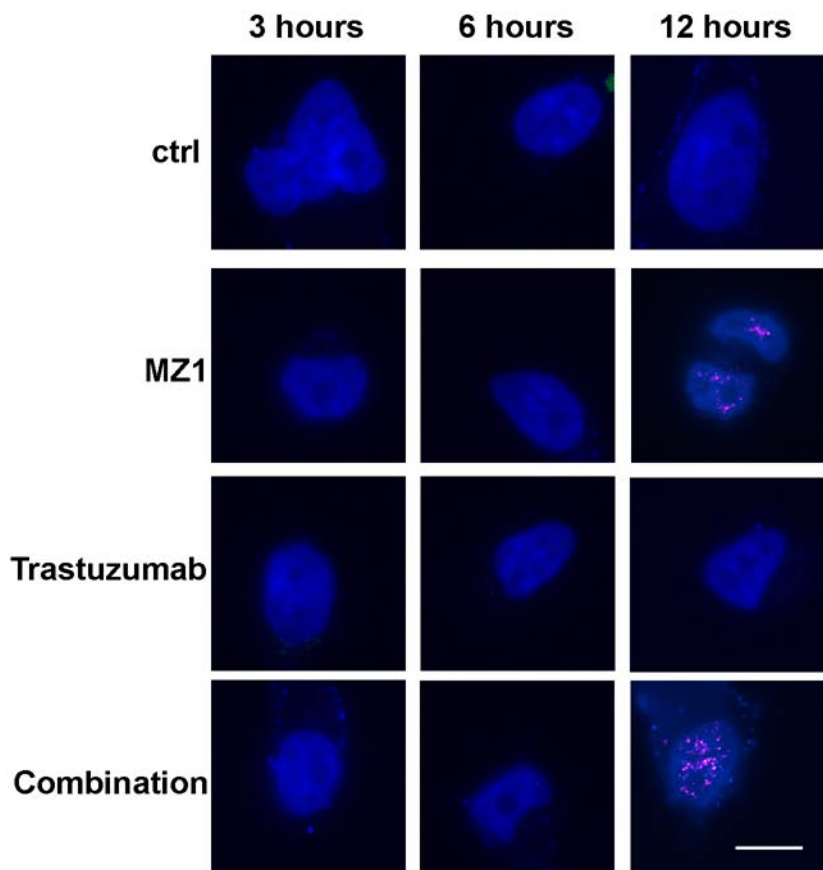

Supplement: Supplementary file 3 — Additional file 3: Figure S3. A, Western blot showing expression level in BT474 cells of pH2AX in BT474 cells treated after 3, 6 and 12 h with MZ1 (100 nM), trastuzumab (10 nM), or the MZ1-trastuzumab combination, or left untreated. B, Fluorescence images of BT474 cells showing pH2AX immunoreactivity (magenta) and DNA staining (blue) obtained by epifluorescence microscopy after MZ1 (100 nM), trastuzumab (10 nM) or the MZ1-trastuzumab combination treatments at the same time points. Scale bar = 10 μm. [file 13046_2021_1907_MOESM3_ESM.pdf]

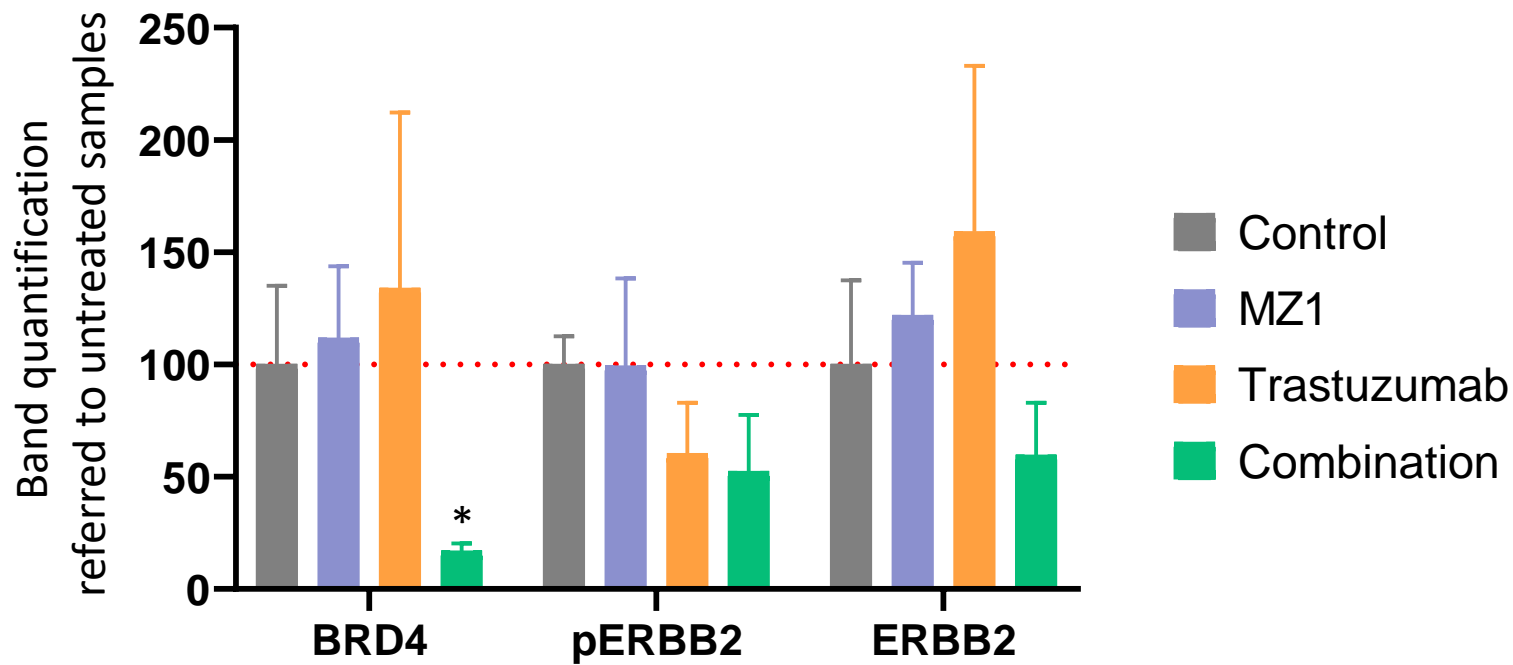

Supplement: Supplementary file 4 — Additional file 4: Figure S4. Quantification of the bands detected in Western blot analysis from Fig. 3b. Densitometry was measured with ImageJ software. BRD4, pERBB2 and ERBB2 bands were normalized to their respective loading controls. Bar graphs are referred to the non-treated samples. *p < 0.05. [file 13046_2021_1907_MOESM4_ESM.pdf]

A

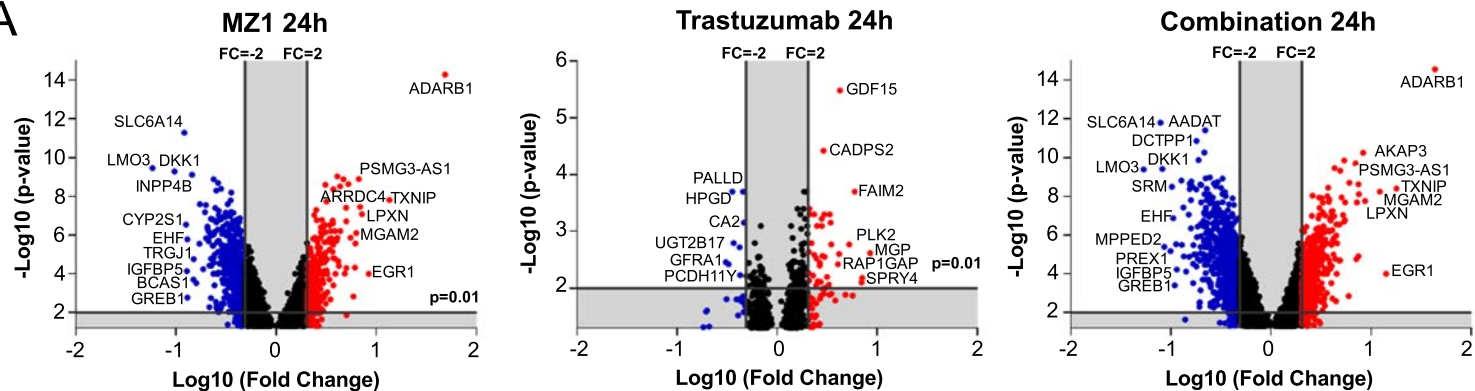

B

## Downregulated genes (24 hours)

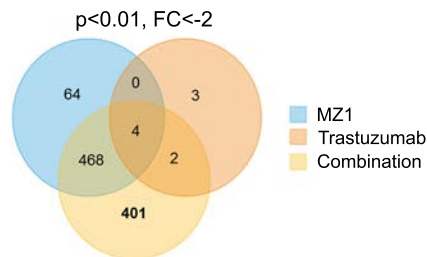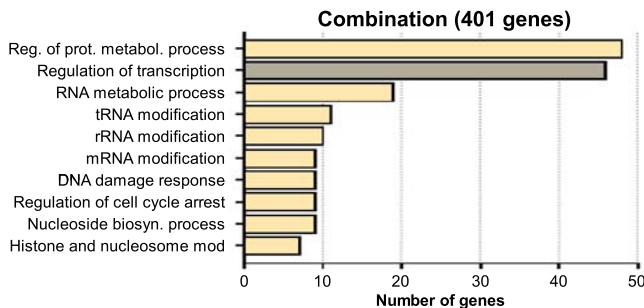

C

## Upregulated genes (24 hours)

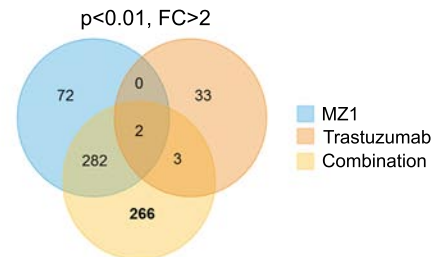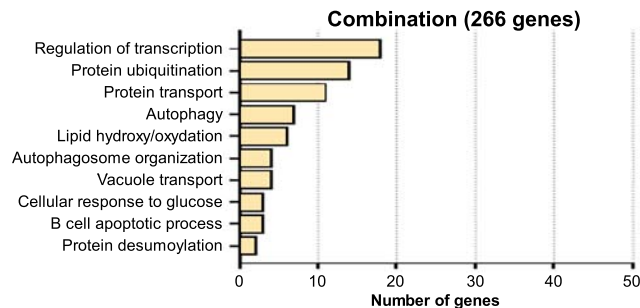

Supplement: Supplementary file 5 — Additional file 5: Figure S5. Analysis of a transcriptomic array in cells treated with MZ1, Trastuzumab or MZ1-Trastuzumab (24 h). A, Volcano plots (p-value (−log 10) vs Fold Change (log10)) of BT474 cells gene expression profile after 24 h treatment with MZ1, trastuzumab, and combination. Upregulated genes (FC > 2) are show in red and downregulated genes (FC < -2) are show in blue. B, Venn diagram showing the number of downregulated genes in each treatment using p-value< 0.01 and FC < -2 as threshold. Bottom bar graph, functional analyses of the 401 altered genes after combination treatment using EnrichR Online Tool. Biological process gene ontologies (p < 0.01) grouped are shown. C, Venn diagram showing the number of upregulated genes in each treatment condition using p-value < 0.01 and FC < -2 as threshold. Bottom bar graph, functional analyses of the 266 altered genes after combination treatment using EnrichR Online Tool. Biological process gene ontologies (p < 0.01) grouped are shown. [file 13046_2021_1907_MOESM5_ESM.pdf]

# B

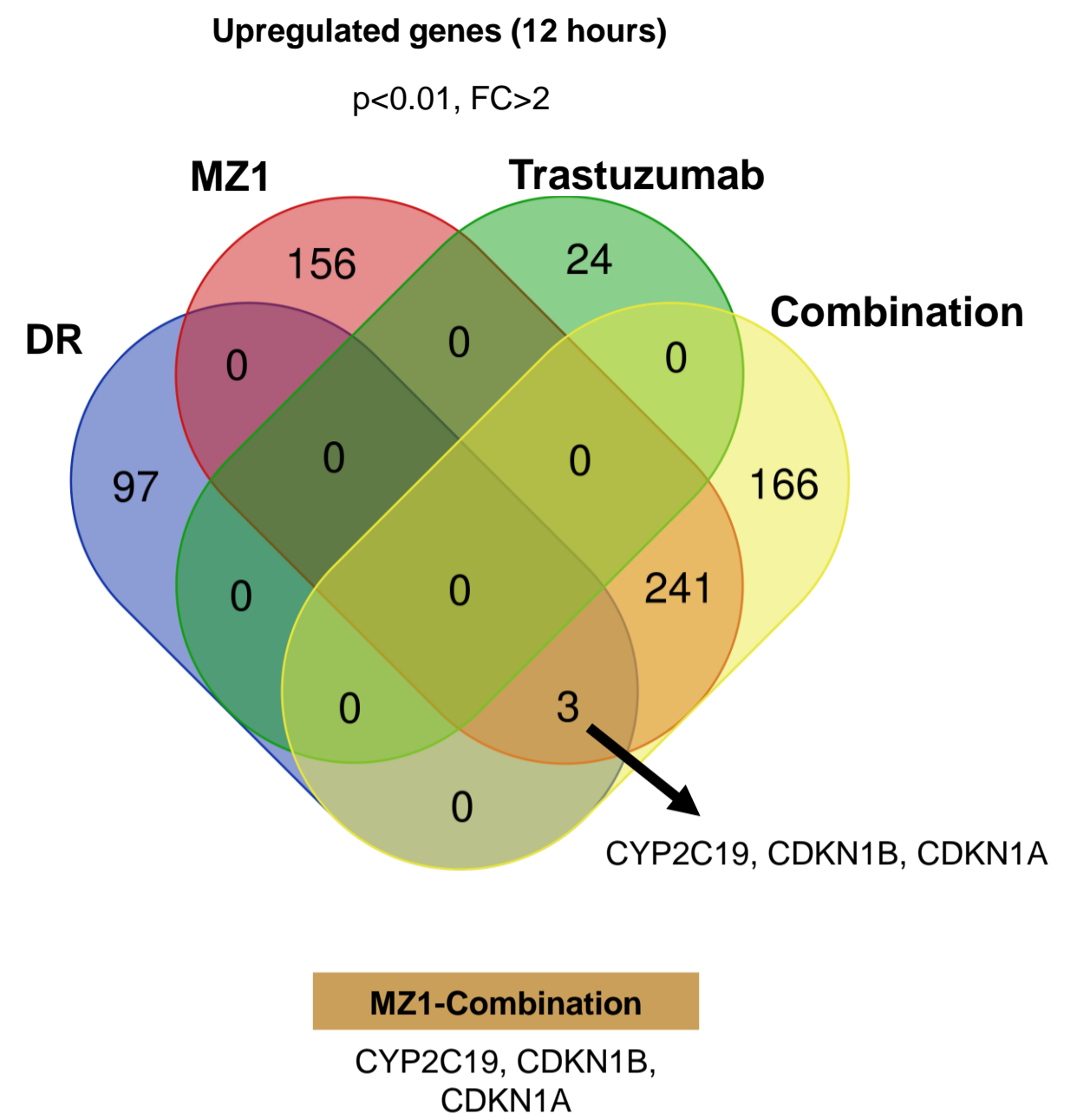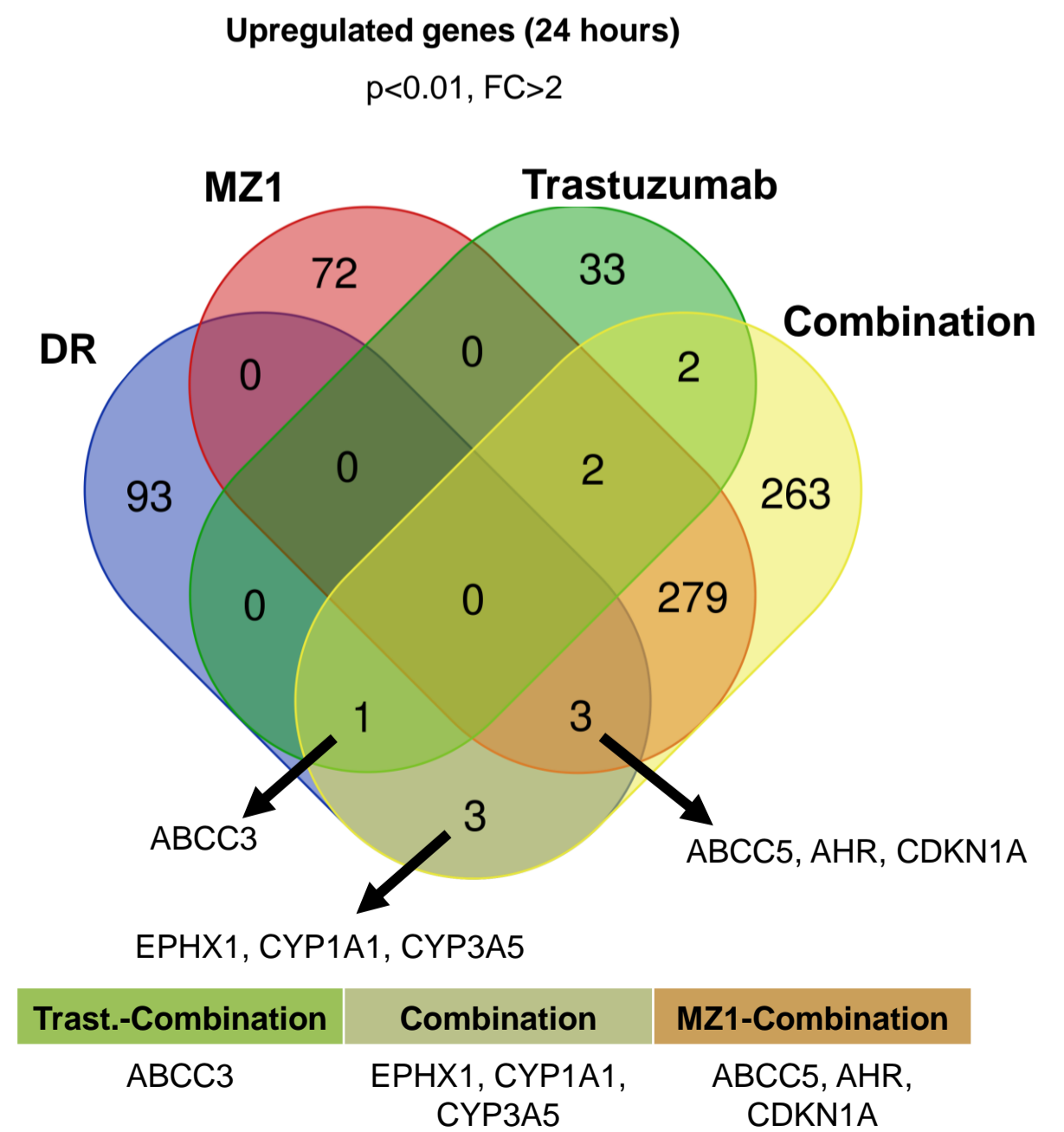

Supplement: Supplementary file 7 — Additional file 7: Figure S7. Venn diagram showing the number of downregulated or upregulated genes (A, 12 h and B, 24 h) in each treatment condition and a drug resistance gene list (DR) (NCBI, Accession: GPL1124). DR and each condition overlapping genes are specified. [file 13046_2021_1907_MOESM7_ESM.pdf]
