# Supplementary material for: MZ1 co-operates with trastuzumab in HER2 positive breast cancer
Source: J Exp Clin Cancer Res. 2021 Mar 19;40:106. doi: 10.1186/s13046-021-01907-9 (PMC7980639; doi:10.1186/s13046-021-01907-9)
Supplement: Supplementary file 6 — Additional file 6: Figure S6. Venn diagram showing the number of downregulated or upregulated genes (A, 12 h and B, 24 h) in MZ1 and combination treatment condition. Functional analyses of the altered genes after each treatment using EnrichR Online Tool are shown. Biological process gene ontologies (p < 0.01) grouped are shown. Genes were classified in those specifically modified after treatments with MZ1 or combination, and those commonly shared. Results as presented as percentage of total genes of each biological process. [file 13046_2021_1907_MOESM6_ESM.pdf]

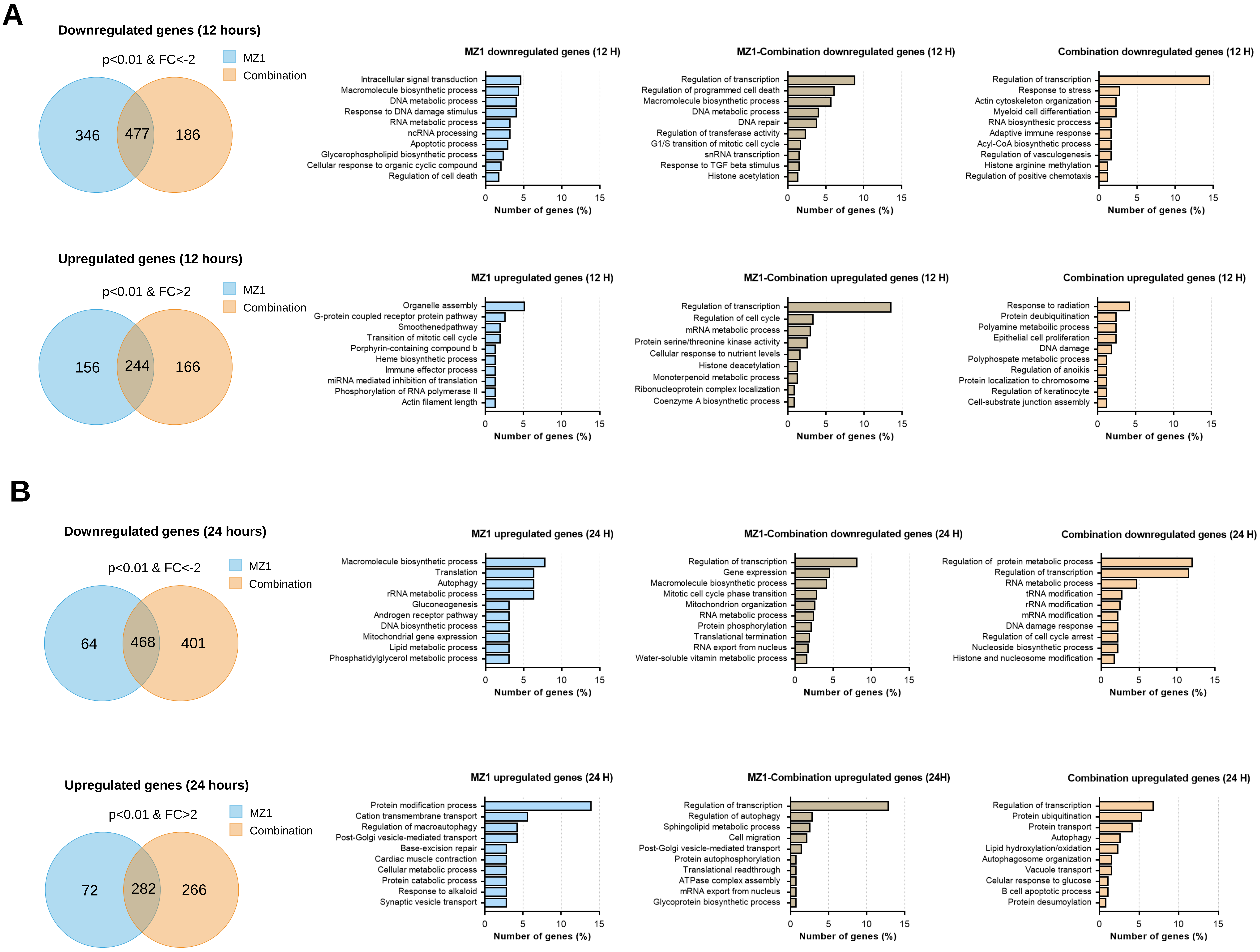

A
Downregulated genes (12 hours)
p<0.01 & FC<-2
MZ1
Combination
346
477
186
MZ1 downregulated genes (12 H)
Intracellular signal transduction
Macromolecule biosynthetic process
DNA metabolic process
Response to DNA damage stimulus
RNA metabolic process
ncRNA processing
Apoptotic process
Glycerophospholipid biosynthetic process
Cellular response to organic cyclic compound
Regulation of cell death
Number of genes (%)
MZ1-Combination downregulated genes (12 H)
Regulation of transcription
Regulation of programmed cell death
Macromolecule biosynthetic process
DNA metabolic process
DNA repair
Regulation of transferase activity
G1/S transition of mitotic cell cycle
snRNA transcription
Response to TGF beta stimulus
Histone acetylation
Number of genes (%)
Combination downregulated genes (12 H)
Regulation of transcription
Response to stress
Actin cytoskeleton organization
Myeloid cell differentiation
RNA biosynthesic proccess
Adaptive immune response
Acyl-CoA biosynthetic process
Regulation of vasculogenesis
Histone arginine methylation
Regulation of positive chemotaxis
Number of genes (%)
Upregulated genes (12 hours)
p<0.01 & FC>2
MZ1
Combination
156
244
166
MZ1 upregulated genes (12 H)
Organelle assembly
G-protein coupled receptor protein pathway
Smoothenedpathway
Transition of mitotic cell cycle
Porphyrin-containing compound b
Heme biosynthetic process
Immune effector process
miRNA mediated inhibition of translation
Phosphorylation of RNA polymerase II
Actin filament length
Number of genes (%)
MZ1-Combination upregulated genes (12 H)
Regulation of transcription
Regulation of cell cycle
mRNA metabolic process
Protein serine/threonine kinase activity
Cellular response to nutrient levels
Histone deacetylation
Monoterpenoid metabolic process
Ribonucleoprotein complex localization
Coenzyme A biosynthetic process
Number of genes (%)
Combination upregulated genes (12 H)
Response to radiation
Protein deubiquitination
Polyamine metabolic process
Epithelial cell proliferation
DNA damage
Polyphospate metabolic process
Regulation of anoikis
Protein localization to chromosome
Regulation of keratinocyte
Cell-substrate junction assembly
Number of genes (%)
B
Downregulated genes (24 hours)
p<0.01 & FC<-2
MZ1
Combination
64
468
401
MZ1 upregulated genes (24 H)
Macromolecule biosynthetic process
Translation
Autophagy
rRNA metabolic process
Gluconeogenesis
Androgen receptor pathway
DNA biosynthetic process
Mitochondrial gene expression
Lipid metabolic process
Phosphatidylglycerol metabolic process
Number of genes (%)
MZ1-Combination downregulated genes (24 H)
Regulation of transcription
Gene expression
Macromolecule biosynthetic process
Mitotic cell cycle phase transition
Mitochondrion organization
RNA metabolic process
Protein phosphorylation
Translational termination
RNA export from nucleus
Water-soluble vitamin metabolic process
Number of genes (%)
Combination downregulated genes (24 H)
Regulation of protein metabolic process
Regulation of transcription
RNA metabolic process
tRNA modification
rRNA modification
mRNA modification
DNA damage response
Regulation of cell cycle arrest
Nucleoside biosynthetic process
Histone and nucleosome modification
Number of genes (%)
Upregulated genes (24 hours)
p<0.01 & FC>2
MZ1
Combination
72
282
266
MZ1 upregulated genes (24 H)
Protein modification process
Cation transmembrane transport
Regulation of macroautophagy
Post-Golgi vesicle-mediated transport
Base-excision repair
Cardiac muscle contraction
Cellular metabolic process
Protein catabolic process
Response to alkaloid
Synaptic vesicle transport
Number of genes (%)
MZ1-Combination upregulated genes (24H)
Regulation of transcription
Regulation of autophagy
Sphingolipid metabolic process
Cell migration
Post-Golgi vesicle-mediated transport
Protein autophosphorylation
Translational readthrough
ATPase complex assembly
mRNA export from nucleus
Glycoprotein biosynthetic process
Number of genes (%)
Combination upregulated genes (24 H)
Regulation of transcription
Protein ubiquitination
Protein transport
Autophagy
Lipid hydroxylation/oxidation
Autophagosome organization
Vacuole transport
Celular response to glucose
B cell apoptotic process
Protein desumoylation
Number of genes (%)
