# Supplementary material for: MZ1 co-operates with trastuzumab in HER2 positive breast cancer
Source: J Exp Clin Cancer Res. 2021 Mar 19;40:106. doi: 10.1186/s13046-021-01907-9 (PMC7980639; doi:10.1186/s13046-021-01907-9)
Supplement: Supplementary file 8 — Additional file 8: Table S1. List of primary human monoclonal/polyclonal antibodies. Table S2. Primer sequences used for HOXB7, MEIS2, TCERG1, and, DNAJC2 genes qPCR amplification. Table S3. Genes selection related to regulation of transcription process. Prognosis value of regulation of transcription genes altered in combination for 12 h, 24 h, or both. Selected genes using a threshold of HR > 1, p-value< 0.05, and FDR-value< 20% are highlighted in grey. [file 13046_2021_1907_MOESM8_ESM.pptx]

## Slide 1
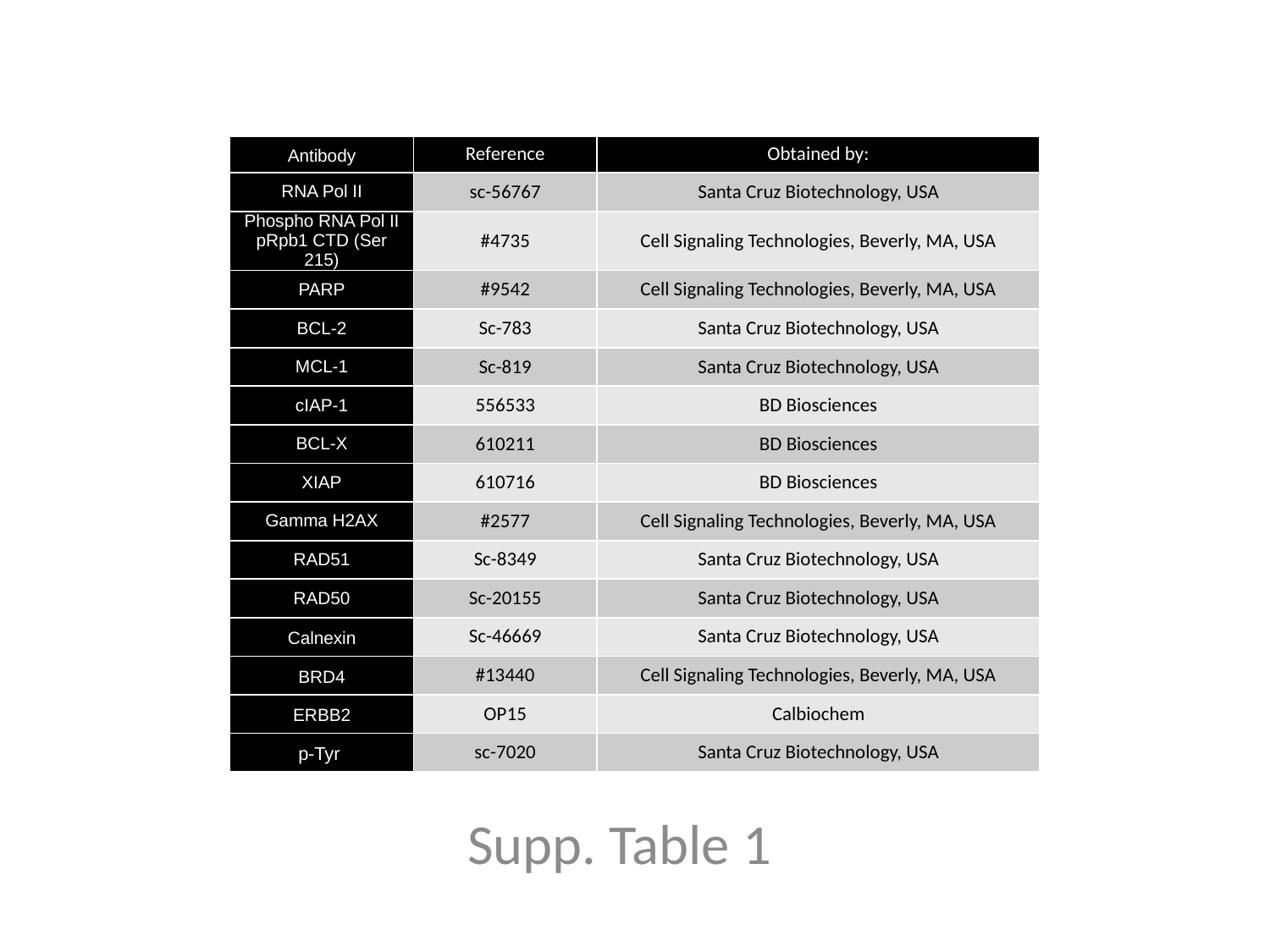

| Antibody | Reference | Obtained by: |
| --- | --- | --- |
| RNA Pol II | sc-56767 | Santa Cruz Biotechnology, USA |
| Phospho RNA Pol II pRpb1 CTD (Ser 215) | #4735 | Cell Signaling Technologies, Beverly, MA, USA |
| PARP | #9542 | Cell Signaling Technologies, Beverly, MA, USA |
| BCL-2 | Sc-783 | Santa Cruz Biotechnology, USA |
| MCL-1 | Sc-819 | Santa Cruz Biotechnology, USA |
| cIAP-1 | 556533 | BD Biosciences |
| BCL-X | 610211 | BD Biosciences |
| XIAP | 610716 | BD Biosciences |
| Gamma H2AX | #2577 | Cell Signaling Technologies, Beverly, MA, USA |
| RAD51 | Sc-8349 | Santa Cruz Biotechnology, USA |
| RAD50 | Sc-20155 | Santa Cruz Biotechnology, USA |
| Calnexin | Sc-46669 | Santa Cruz Biotechnology, USA |
| BRD4 | #13440 | Cell Signaling Technologies, Beverly, MA, USA |
| ERBB2 | OP15 | Calbiochem |
| p-Tyr | sc-7020 | Santa Cruz Biotechnology, USA |
Supp. Table 1

## Slide 2
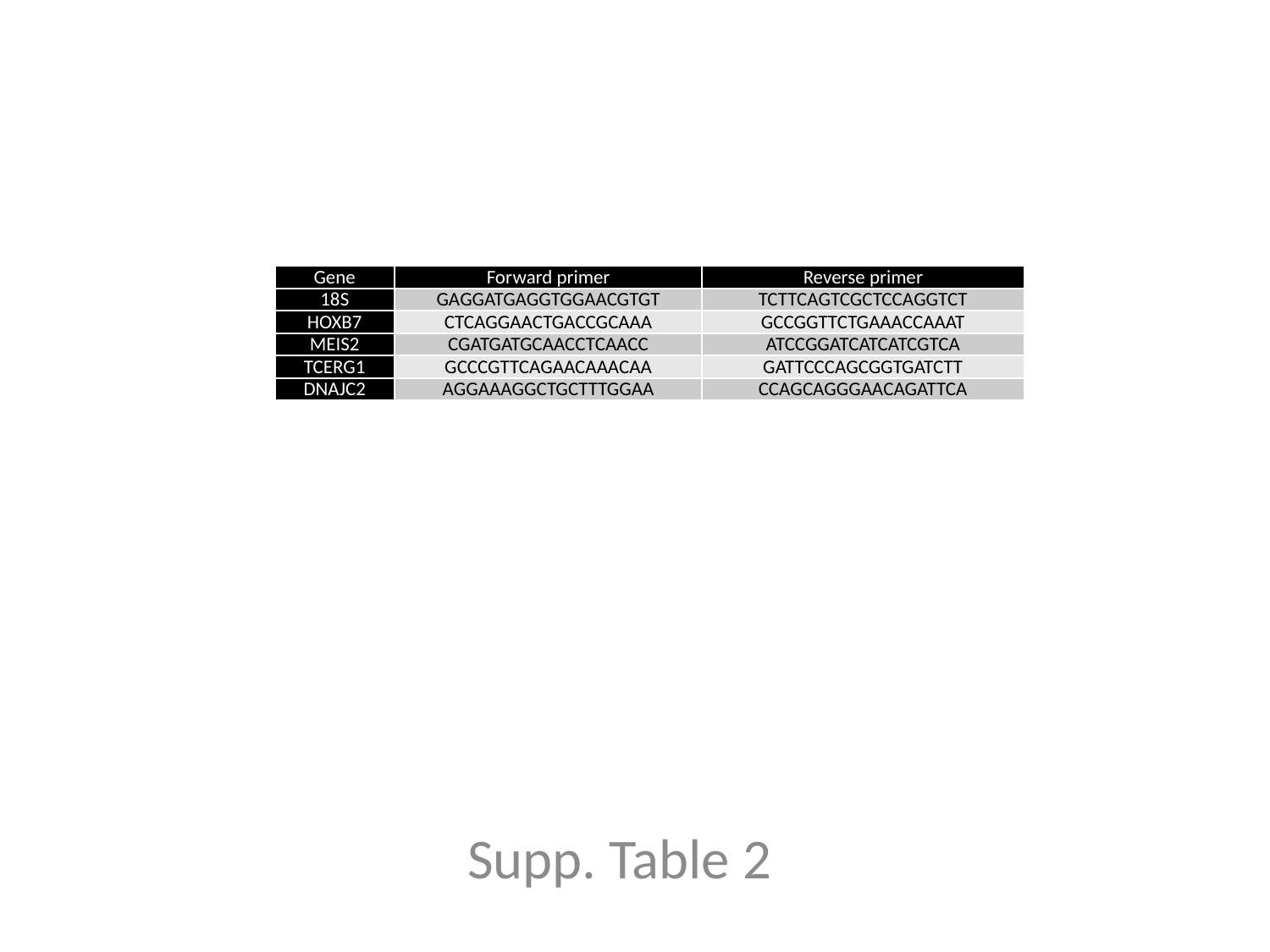

| Gene | Forward primer | Reverse primer |
| --- | --- | --- |
| 18S | GAGGATGAGGTGGAACGTGT | TCTTCAGTCGCTCCAGGTCT |
| HOXB7 | CTCAGGAACTGACCGCAAA | GCCGGTTCTGAAACCAAAT |
| MEIS2 | CGATGATGCAACCTCAACC | ATCCGGATCATCATCGTCA |
| TCERG1 | GCCCGTTCAGAACAAACAA | GATTCCCAGCGGTGATCTT |
| DNAJC2 | AGGAAAGGCTGCTTTGGAA | CCAGCAGGGAACAGATTCA |
Supp. Table 2

## Slide 3
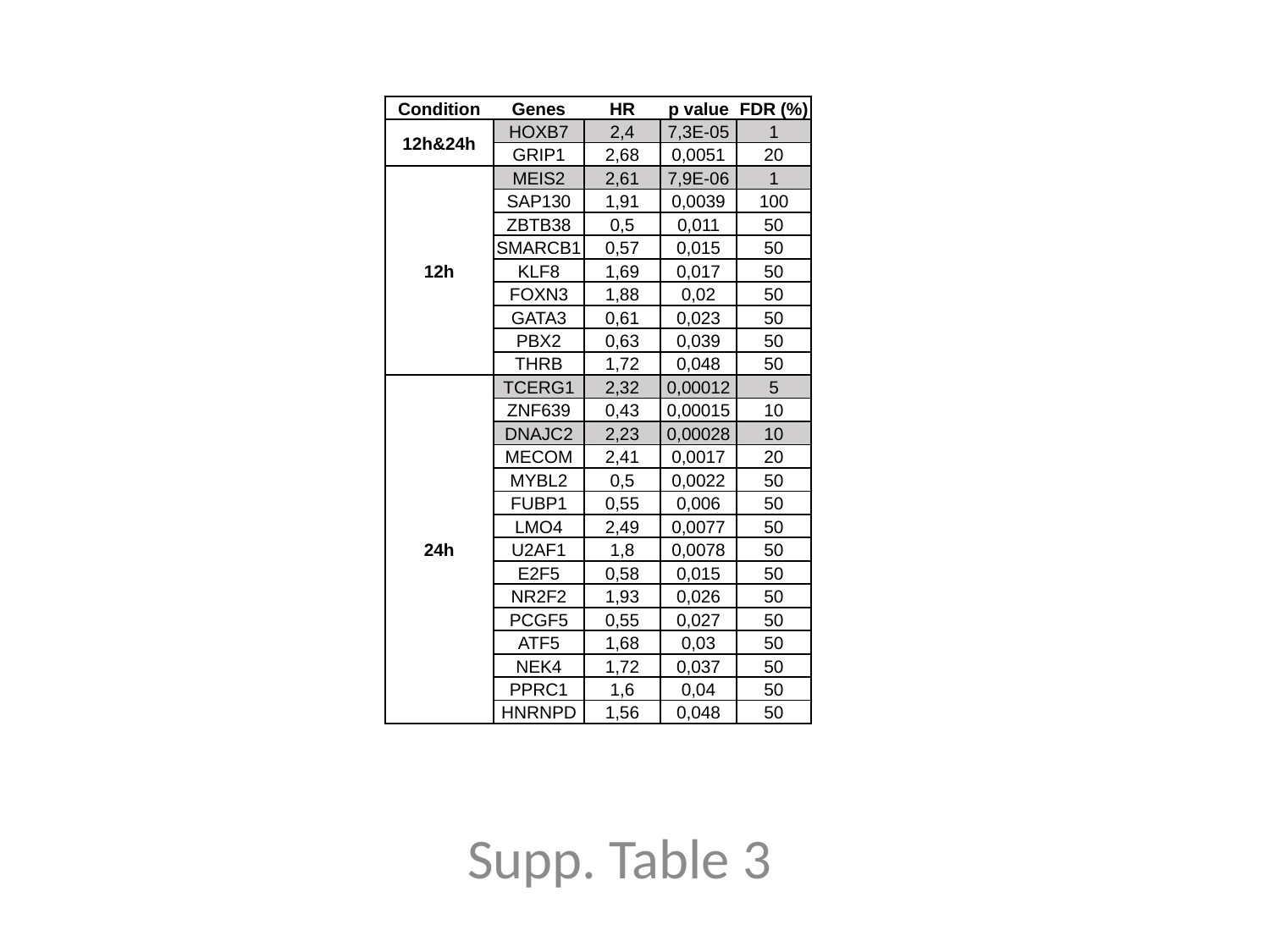

| Condition | Genes | HR | p value | FDR (%) |
| --- | --- | --- | --- | --- |
| 12h&24h | HOXB7 | 2,4 | 7,3E-05 | 1 |
| | GRIP1 | 2,68 | 0,0051 | 20 |
| 12h | MEIS2 | 2,61 | 7,9E-06 | 1 |
| | SAP130 | 1,91 | 0,0039 | 100 |
| | ZBTB38 | 0,5 | 0,011 | 50 |
| | SMARCB1 | 0,57 | 0,015 | 50 |
| | KLF8 | 1,69 | 0,017 | 50 |
| | FOXN3 | 1,88 | 0,02 | 50 |
| | GATA3 | 0,61 | 0,023 | 50 |
| | PBX2 | 0,63 | 0,039 | 50 |
| | THRB | 1,72 | 0,048 | 50 |
| 24h | TCERG1 | 2,32 | 0,00012 | 5 |
| | ZNF639 | 0,43 | 0,00015 | 10 |
| | DNAJC2 | 2,23 | 0,00028 | 10 |
| | MECOM | 2,41 | 0,0017 | 20 |
| | MYBL2 | 0,5 | 0,0022 | 50 |
| | FUBP1 | 0,55 | 0,006 | 50 |
| | LMO4 | 2,49 | 0,0077 | 50 |
| | U2AF1 | 1,8 | 0,0078 | 50 |
| | E2F5 | 0,58 | 0,015 | 50 |
| | NR2F2 | 1,93 | 0,026 | 50 |
| | PCGF5 | 0,55 | 0,027 | 50 |
| | ATF5 | 1,68 | 0,03 | 50 |
| | NEK4 | 1,72 | 0,037 | 50 |
| | PPRC1 | 1,6 | 0,04 | 50 |
| | HNRNPD | 1,56 | 0,048 | 50 |
Supp. Table 3
